# Hot shape transformation: the role of PSar dehydration in stomatocyte morphogenesis

**DOI:** 10.3762/bjoc.21.5

**Published:** 2025-01-08

**Authors:** Remi Peters, Levy A Charleston, Karinan van Eck, Teun van Berlo, Daniela A Wilson

**Affiliations:** 1 Institute of Molecules and Materials, Radboud University, Heyendaalseweg 135, 6525 AJ, Nijmegen, The Netherlands

**Keywords:** biodegradable, poly(benzyl glutamate), polysarcosine, shape transformation, stomatocyte, supramolecular chemistry

## Abstract

Polysarcosine emerges as a promising alternative to polyethylene glycol (PEG) in biomedical applications, boasting advantages in biocompatibility and degradability. While the self-assembly behavior of block copolymers containing polysarcosine-containing polymers has been reported, their potential for shape transformation remains largely untapped, limiting their versatility across various applications. In this study, we present a comprehensive methodology for synthesizing, self-assembling, and transforming polysarcosine-poly(benzyl glutamate) block copolymers, resulting in the formation of bowl-shaped vesicles, disks, and stomatocytes. Under ambient conditions, the shape transformation is restricted to bowl-shaped vesicles due to the membrane's flexibility and permeability. However, dehydration of the polysarcosine broadens the possibilities for shape transformation. These novel structures exhibit asymmetry and possess the capability to encapsulate smaller structures, thereby broadening their potential applications in drug delivery and nanotechnology. Our findings shed light on the unique capabilities of polysarcosine-based polymers, paving the way for further exploration and harnessing of their distinctive properties in biomedical research.

## Introduction

Polymeric vesicles represent a promising candidate for usage in drug delivery systems due to their facile assembly and ability to provide a stable soft interface. Among these materials, polyethylene glycol-polystyrene block copolymers (PEG-PS) stand out for their versatility and adaptability. These copolymers exhibit a remarkable propensity for self-assembly, allowing the formation of vesicles capable of undergoing diverse shape transformations. Notably, they can adopt the distinctive stomatocyte morphology, characterized by a concave shape with a central cavity [1]. Such structures hold significant potential for drug delivery applications as nanomotors, offering both encapsulation capability and controlled release functionalities [2–3]. Moreover, researchers have explored novel shapes utilizing PEG-PS copolymers, including the growth of protrusions from the vesicle surface [4]. While these emerging shapes warrant further investigation to elucidate their optimal utilities, their creation underscores the material's versatility in shape manipulation. This ability to engineer a spectrum of shapes from a single material holds profound implications for drug delivery and beyond. By tailoring vesicle morphology to specific requirements, researchers can optimize drug encapsulation, targeting, and release, advancing the efficacy and precision of therapeutic interventions. Continued exploration of these versatile materials promises to unlock new avenues in pharmaceuticals and biomedical engineering. The problem here is that PEG-PS is a non-biodegradable material and this poses significant challenges for its use in drug delivery applications, particularly concerning potential accumulation in the body [5]. Recent advancements have sought to address this issue by replacing the hydrophobic block with biodegradable alternatives, like polylactic acid, resulting in PEG-PDLLA block copolymers capable of forming vesicles that are able to undergo shape transformation towards stomatocytes [6]. Despite this progress, the persistence of the non-degradable PEG segment is still ongoing as PEG is regarded as the benchmark for hydrophilic polymers used in drug delivery [7].

The non-biodegradability of PEG under most conditions, coupled with recently discovered immunogenic responses to it, has led to increasing concerns [8–9]. This debate has prompted exploration into novel materials that resemble PEG, retaining its positive attributes, but add biodegradability. Two of such classes of polymers that would form viable alternatives are polyoxazolines and polypeptides [10–11], both of which possess biodegradable and biocompatible properties. Moreover, these materials offer versatility in their synthesis, allowing for the incorporation of various building blocks to tailor the polymers to desired specifications. Additionally, they lend themselves well to the synthesis of block copolymers, further expanding their potential applications for the use in drug delivery systems [12].

In this regard, synthetic polypeptides emerge as a promising candidate for constructing biodegradable and biocompatible polymersomes. Leveraging the inherent presence of peptide-degrading mechanisms within the human body and the versatile chemical functionalities of naturally occurring amino acids, synthetic polypeptides offer a robust platform for designing drug delivery systems that meet the criteria of biodegradability and biocompatibility [13]. The present study focuses on a polysarcosine and poly-ʟ-(benzyl glutamate) block copolymer (PSar-PBLG), as it is known to be able to self-assemble in a variety of structures, including micelles, vesicles, and nanoparticles [14–15]. However, the versatility of these polymers in shaping vesicles into asymmetric structures suitable for nanomotors remains relatively unexplored. While previous research has demonstrated their ability to form round vesicles and some tubular shapes, the pursuit of asymmetric structures, such as the stomatocyte, presents a novel challenge and opportunity in the field [16–17].

The stomatocyte shape would be an excellent addition because of its demonstrated suitability for nanomotor fabrication [18]. The necessary shape transformation for achieving this morphology is primarily driven by changes in osmotic pressure. Initially achieved through dialysis and later by the addition of PEG solution [1,18], the process involves deflating the polymeric vesicle, prompting it to bend and adopt the stomatocyte configuration. Several critical factors contribute to this transformation. Firstly, the application of osmotic pressure which must be sufficiently robust. This control is notably easier with the addition of PEG, as it swiftly creates a substantial osmotic gradient [19]. Secondly, the vesicle's properties are crucial, particularly its permeability. Excessive permeability allows water molecules – and potentially larger molecules – to traverse the membrane without exerting adequate force, undermining the transformation process [20–21]. Lastly, membrane stiffness plays a role; the stiffness of the membrane determines how it responds to the applied force. Besides that stiffness should be sufficient to maintain membrane curvature for a shape transformation to occur [22]. This is particularly important when domains are formed over the membrane with different stiffness which can lead to an asymmetrical response [23–25]. An excessive stiffness will eventually even impede deformation entirely [26]. This characteristic is harnessed in systems to stabilize shapes by water addition, effectively locking the membrane in a kinetically stable state [27].

In a recent study by Elafify, M. S. et al. it was demonstrated that polysarcosine-based self-assemblies could undergo shape transformation via dehydration induced by heating [16]. This dehydration of the polysarcosine caused in their system a complete shape transformation but it would effectively also reduce membrane permeability and enhance osmotic pressure, as temperature is a factor in the van ‘t Hoff formula for osmotic pressure [21]. Additionally, dehydration strengthens polysarcosine chains interactions [16], potentially rendering the membrane too rigid for shape transformation. This means that the heating of the vesicles made with polysarcosine would improve the effectivity of the osmotic pressure applied up to a certain point, after which the membrane would be too rigid for deformation. Within this window it is expected that stomatocytes could be obtained using PSar-PBLG block copolymers.

## Results and Discussion

**Scheme 1:**
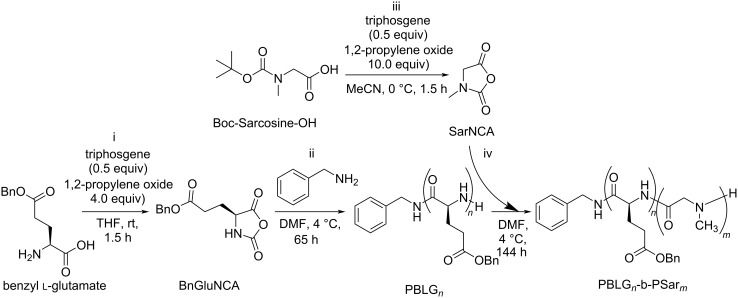
i) Synthesis of benzyl glutamate NCA using phosgene and propylene oxide as a scavenger. ii) Ring-opening polymerization of BnGluNCA using benzylamine as initiator. iii) Synthesis of sarcosine NCA starting from Boc-protected sarcosine using phosgene and propylene oxide as a scavenger. iv) Ring-opening polymerization of Sar NCA using the benzyl glutamate homopolymer as macro initiator.

Starting with the synthesis of the PSar-PBLG that was used for all the following systems, it was found that a polymer with a length of 50 units sarcosine and 40 units benzyl glutamate would be able to form vesicles between 300–700 nm (Supporting Information File 1, Figure S22). The synthesis of PSar-PBLG (Scheme 1) involved anionic ring-opening polymerization of *N*-carboxyanhydrides (NCAs), following a protocol adapted from Tian et al. [28]. High-purity monomers were obtained using this method, ensuring the subsequentially polymer synthesis proceeded with minimal impurities. Sequential polymerization commenced with poly(benzyl glutamate) (PBLG) as the hydrophobic block, which was done to prevent ending on a primary amine in the final product which could disrupt self-assembly due to its ionizable nature. Subsequently, PBLG served as a macroinitiator for the polymerization of sarcosine NCA, yielding the final polymer with high yield and low polydispersity index (PDI), crucial for effective self-assembly [29].

Previous studies on PEG-PS vesicles demonstrated a solvent-exchange method for vesicle formation, followed by a shape transformation induced by osmotic pressure from PEG addition [18]. The structure was then frozen by addition water – this process was facilitated by the glass-transition temperature (*T*_g_) of the polymer, marking the transition from a fluid to a glassy state. To replicate this methodology, it is important that the *T*_g_ of PSar-PBLG is high enough to freeze the different morphologies by water addition. The *T*_g_ was determined using differential scanning calorimetry (DSC) (Supporting Information File 1, Figure S18). The results revealed a *T*_g_ of approximately 100 °C for PSar-PBLG, indicating the potential for shape locking through transitions between fluidity and a glassy state. Additionally, examination of the thermogram unveiled a peak during the initial heating cycle, spanning from 40 °C to 90 °C (Supporting Information File 1, Figure S18). This peak corresponds with the dehydration temperature of polysarcosine, suggesting its role in facilitating subsequent shape manipulation [16].

**Figure 1:**
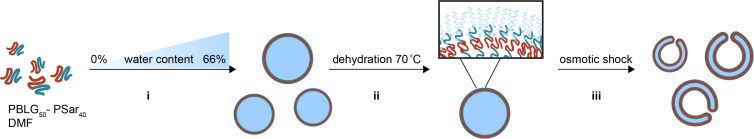
i) The PBLG-PSar block copolymers are dissolved in DMF and then assembled though the solvent-exchange method by addition of 66% water. ii) The membrane is dehydrated at 70 °C for 1 min before starting the shape transformation. iii) The shape transformation is induced by osmotic shock. This is done by the addition of 5 µL PEG_2000_ solution (400 mg/mL). The osmotic shock can also be induced by PSar solution or saccharose solution.

The assembly of the PSar-PBLG block copolymers was optimally achieved by dissolving the block copolymers in DMF followed by a solvent-exchange method. Here, Milli-Q water was gradually introduced to form monodisperse polymersomes (Figure 1i, and Supporting Information File 1, Figure S22). Hexafluoroisopropanol (HFIP) emerged as another solvent yielding monodisperse assemblies. HFIP promotes formation of alpha helices in peptides and this property yielded vesicles with different morphologies [30]. The resulting vesicles looked darker compared to those formed in DMF, as observed through TEM (Supporting Information File 1, Figure S21).

For the shape transformation experiments only the vesicles assembled in DMF were used. To validate the structure of the self-assembly, cryo-TEM was conducted, revealing that the obtained vesicles exhibited a wrinkled appearance, differing from the typically round vesicles observed in systems like PEG-PS [20] or PEG-PDLLA [31]. This wrinkled morphology suggests that the membrane may possess greater flexibility compared to the aforementioned systems (Supporting Information File 1, Figure S22). This effect is more often observed in liposome systems that have thinner and more flexible membranes. In this example by Buscema et al. it was observed that liposome vesicles can deform under mechanical stress to yield similar structures as the PSar-PBLG vesicles [32].

It was attempted to change the shape of the assembled vesicles following a similar method as previously developed for PEG-PS systems. Cryo-TEM analysis revealed only a slight decrease in vesicle volume and a somewhat flattened appearance (Supporting Information File 1, Figure S21). Based on our observations and those of others working with similar polymers [16], we deduced that the membrane of PBLG-PSar is overly flexible and permeable to water, resulting in a minimal shape transformation. The osmotic pressure exerted on a membrane depends on its permeability to water, with PBLG-PSar experiencing significantly less force on the membrane compared to PEG-PS systems, even with the same amount of added PEG. Simply increasing the solute concentration proved ineffective; an alternative approach was needed.

Examining the van 't Hoff equation for osmotic pressure, Π = i*MRT*, four factors play significant roles: solute concentration *M* (in this case, PEG), the van 't Hoff constant *i* (which is 1 for non-dissociating molecules), temperature *T*, and the ideal gas constant *R*. Increasing the temperature can enhance osmotic pressure, similar to solute concentration. This equation does not directly include membrane permeability as it only relates the osmotic pressure to temperature and concentration. However, membrane permeability indirectly affects osmotic pressure difference over the membrane. Permeability influences the concentration gradient of solute particles across the membrane [21], meaning that in this case membrane permeability affects the osmotic pressure that can be exerted on the membrane. Dehydrating polysarcosine by raising the temperature influences most of these parameters, reducing permeability and increasing membrane stiffness due to enhanced sarcosine chain interactions [16].

**Figure 2:**
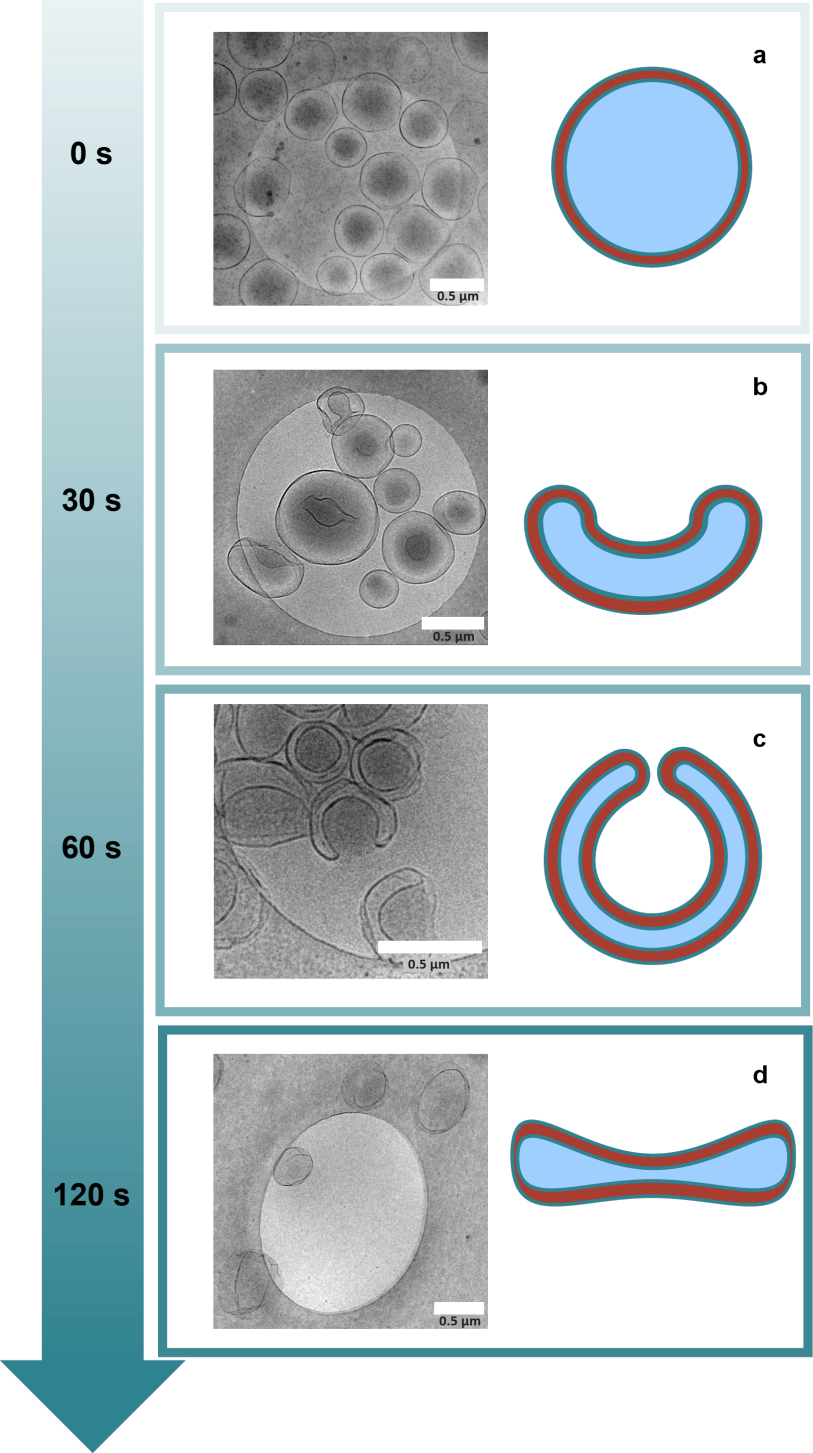
Progression of shape transformation of PSar-PBLG vesicles at 70 °C (scalebar 0.5 µm). a) Initial (0 s): polymersome formation. b) Transition (30 s): formation of a vesicle with a dent and stomatocytes. c) Further shape transition (60 s): to a wide-open stomatocyte. d) Final (120 s, angle 35°): shape evolved into a disk after 120 s before quenching.

Heating the sample to 70 °C and then applying osmotic shock through PEG addition, followed by quenching with water, transformed the shape into a stomatocyte as demonstrated in Figure 1. The progression of shape transformation was monitored from 0 to 120 seconds before quenching. Initially, upon heating and immediate quenching, typical vesicles were observed. This contrasted with earlier observations of wrinkled membranes, suggesting increased membrane rigidity due to heating (Figure 2a). Within 5 seconds, a slight shape transformation became evident, with most vesicles exhibiting minor dents and some forming stomatocytes (Figure 2b). By the 60 second mark, all shapes had transitioned into stomatocytes (Figure 2c), with further waiting resulting in a transformation towards disk-like structures (Figure 2d). This route for the shape transformation appears to deviate slightly from the conventional method, where a disk is first formed and then symmetrically deflated [20]. This deviation might be due to domain formation during dehydration, which results in more rigid domains. The anomalous shapes observed, with bending starting from seemingly random positions, support this hypothesis (Supporting Information File 1, Figure S36). These irregular shapes are reminiscent of some anomalous shapes observed in red blood cells [25]. It was also demonstrated that the shape transformation can be reversible when enough time is given for the osmotic pressure to equilibrate. In Supporting Information File 1, Figure S34 it is shown that after the shape transformation to the disk the membrane relaxes back to the polymerosome shape after 1 hour sitting at room temperature with an organic solvent content of 33%

**Figure 3:**
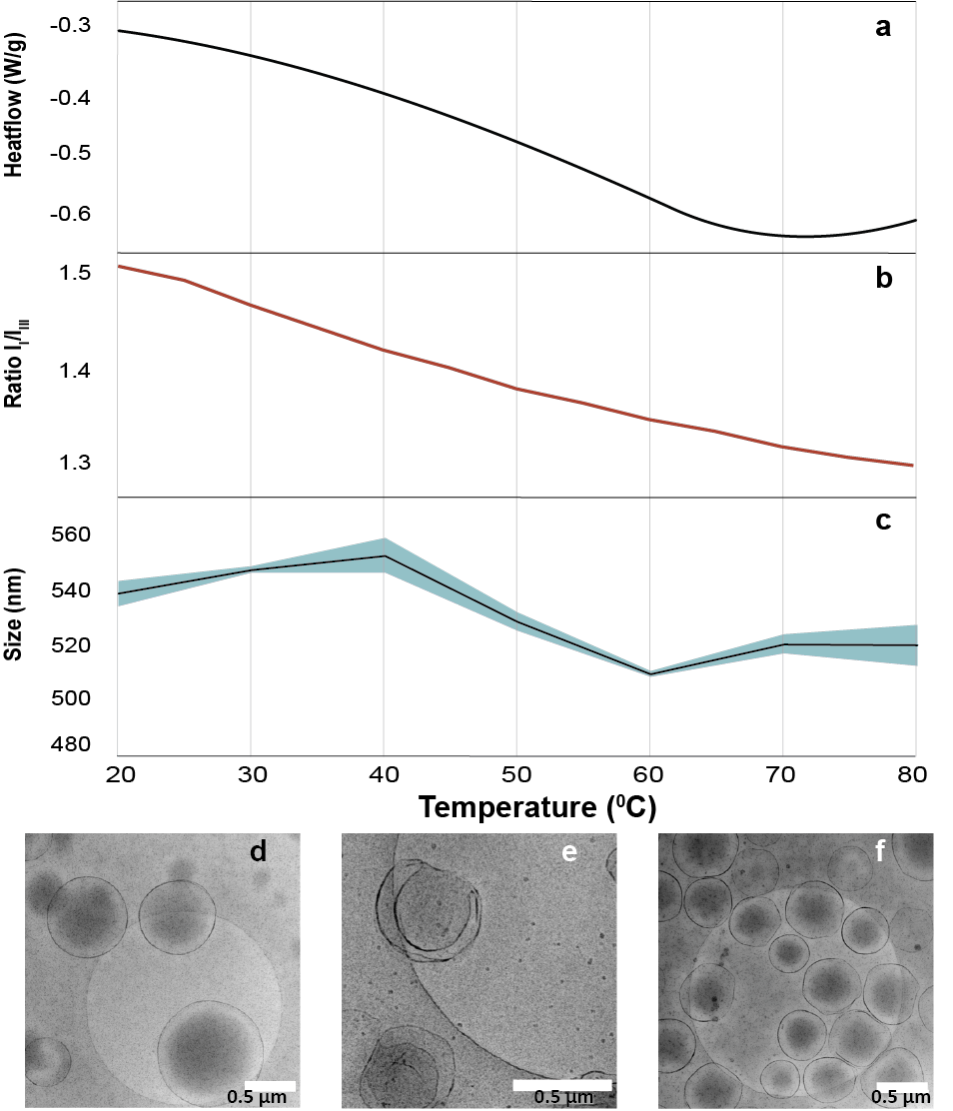
The temperature-dependent behavior of vesicles and shape Transformation: a) Thermograph of PBLG-PSar: Showing dehydration with a peak around 70 °C. b) The fluorescent intensity of pyrene measured at 376.6 nm divided by the intensity at 387.6 nm tracked over temperatures between 20 °C and 80 °C. c) DLS measurement: demonstrating a slight decrease in hydrodynamic radius of the vesicles at higher temperatures. d–f) Vesicles after shape transformation: illustrated at 60 °C, 70 °C, and 80 °C, respectively.

The window for shape transformation seems to lie between 60 °C and 80 °C, with the best result being obtained at 70 °C (Figure 3). When the temperature is too low, the shape transformation cannot happen as the membrane is still too permeable. However, when the membrane gets too stiff, the deformation is prevented regardless of the increased force that is applied.

For further affirmation of the mechanism circular dichroism (CD) spectroscopy was employed to give more insight on the morphological changes in the polymers secondary structure (Supporting Information File 1, Figure S35). A slight decrease in signal intensity corresponding to the alpha helix region was observed [33], indicating a minor disordering of the hydrophobic part potentially aiding flexibility for shape transformation. Additionally, the hydrodynamic radius of vesicles decreased slightly during the heating cycle, indicative of membrane dehydration (Figure 3c). Further proof for the changing membrane properties was shown by a fluorescence measurement of pyrene-PEG-OH in the presence of the PSar-PBLG vesicles. Pyrene is well-known for its sensitivity to environmental hydrophobicity changes [34]. Specifically, the intensity of the I1 peak at 376.6 nm responds significantly to the surrounding polarity, while the I3 peak at 387.6 nm remains relatively stable. The ratio of these peaks (I1/I3) serves as an indicator of the hydrophobicity of the environment, with a lower I1/I3 ratio signifying a more hydrophobic milieu [35]. The pyrene derivative used in this study is predominantly localized in the outer regions of the hydrophilic corona, as demonstrated by the work of Zhang and colleagues [36]. With the increase in temperature there was a slight change in hydrophobicity of the PSar corona in which the pyrene resided. This change shifted the ratio between I1/I3 of pyrenes fluorescence, going down from 1.50 to 1.31 (Figure 3b). This is a clear indication of the environment of the pyrene probe becoming more hydrophobic. The information obtained from DSC, accompanied by the shape transformation experiments are indicative of a clear correlation between the dehydration of the membrane and its susceptibility to change shape through osmotic shock (Figure 3). Besides this, one final evidence for dehydration of the PSar corona was provided by NMR in Supporting Information File 1, Figures S13 and S14 showing a signal decrease with increased temperature, demonstrating a decrease of interaction with the solvent D_2_O.

After replacing PEG with PSar in the self-assembled structures we also show that the osmotic pressure can be induced by PSar in similar fashion as with PEG. Hence, solutions of PSar homopolymer and saccharose were employed, initiating the shape transformation as well (Supporting Information File 1, Figures S33 and S35). This suggests that various hydrophilic biocompatible additives could be viable for inducing osmotic shock in shape transformation. Both PEG and PSar are partially dehydrated at 70 °C making them less effective for usage at high temperatures. Another compound was proposed to induce osmotic pressure, namely saccharose.

The dehydration temperature for saccharose is around 100 °C, indicating that its hydrophilicity remains high compared to PEG and PSar, which have lower dehydration temperatures [37]. Due to this higher temperature, sucrose is expected to induce a greater osmotic shock. This is evident in the results of shape transformation experiments, where more complex structures such as stomatocyte-in-stomatocyte shapes were observed. This suggests that shape transformations triggered by saccharose are more effective at higher temperatures [38].

## Conclusion

Self-assembled vesicles crafted from polysarcosine-poly(benzyl glutamate) block copolymers exhibit distinct properties compared to conventional systems like PEG-PS or PEG-PDLLA, notably their inability to undergo shape transformation though osmotic pressure under conventional conditions. This limitation arises from the inherent membrane flexibility and heightened permeability of these vesicles. However, polysarcosine exhibits a dehydration temperature between 40 °C and 90 °C, triggering the release of water molecules from the hydrophilic layer. Consequently, enhanced interactions between polysarcosine chains ensue, culminating in a more rigid and less permeable membrane structure at an optimum dehydration at 70 °C.

This augmented membrane permeability facilitates the buildup of stronger osmotic pressure within the vesicle, driving deformation into a stomatocyte shape. This novel morphology for polysarcosine-based polymers opens new avenues for utilizing fully biodegradable polymers as nanomotors, leveraging enzyme encapsulation techniques. Moreover, it was observed that a temperature that is too high does not yield further shape transformation but instead results in the formation of more rigid polymersomes. So, the system only operates accordingly in a narrow window.

Lastly besides the PEG addition method for inducing osmotic pressure most hydrophilic molecules should be able to induce enough osmotic pressure to induce shape transformation. The use of saccharose which has a higher dehydration temperature then PEG or PSar seems to be more effective for use at high temperatures.

In summary, the distinctive properties of polysarcosine-poly(benzyl glutamate) block copolymers pave the way for innovative applications in drug delivery and nanotechnology, marking a significant advancement in the field of biodegradable polymer vesicles. Further exploration into optimizing shape transformation conditions tailored to one’s goal and harnessing the unique capabilities of these vesicles holds promise for future biomedical applications.

## Supporting Information

File 1 Experimental part and additional graphics.

## Data Availability

The data that supports the findings of this study is available from the corresponding author upon reasonable request.
