# Supplementary material for: Hot shape transformation: the role of PSar dehydration in stomatocyte morphogenesis
Source: Beilstein J Org Chem. 2025 Jan 8;21:47–54. doi: 10.3762/bjoc.21.5 (PMC11729680; doi:10.3762/bjoc.21.5)
Supplement: File 1 — Experimental part and additional graphics. [file Beilstein_J_Org_Chem-21-47-s001.pdf]

BEILSTEIN JOURNAL OF ORGANIC CHEMISTRY

Supporting Information

for

Hot shape transformation: the role of PSar dehydration in stomatocyte morphogenesis

Remi Peters, Levy A. Charleston, Karinan van Eck, Teun van Berlo
and Daniela A. Wilson

Beilstein J. Org. Chem. **2025,** *21,* 47–54. doi:10.3762/bjoc.21.5

Experimental part and additional graphics

License and Terms: This is a supporting information file under the terms of the Creative Commons Attribution License (https://creativecommons.org/licenses/by/4.0). Please note that the reuse, redistribution and reproduction in particular requires that the author(s) and source are credited and that individual graphics may be subject to special legal provisions.
The license is subject to the *Beilstein Journal of Organic Chemistry* terms and conditions: (https://www.beilstein-journals.org/bjoc/terms)

Contents

List of abbreviations S2
Materials and instrumentation S3
Experimental S4
Self-assembly and shape transformation S8
Dynamic Light Scattering S12
Circular Dichroism measurements S13
Spectra of NMR spectroscopy, GPC and CD S15
TEM and Cryo-TEM images S21
Behaviour of the membrane study S29
Stability and degradation study S35
References S44

List of abbreviations

BCP	Block copolymer
CD	Circular dichroism spectroscopy
CO_2	Carbon dioxide
(Cryo)TEM	(Cryogenic) tunnelling electron microscopy
D.I.T.	Digital integration time
DLS	Dynamic light scattering
DMF	*N,N*-dimethyl formamide
DSC	Differential scanning calorimetry
GPC	Gel permeation chromatography
HFIP	Hexafluorisopropanol
H_2O	Hydrogen dioxide
MilliQ	Ultra-pure water
NCA	*N*-carboxyanhydride
PBLG	Poly(γ-benzyl ʟ-glutamate)
PBLG-b-PSar	Poly(γ-benzyl ʟ-glutamate)-block-polysarcosine
PEG	Polyethylene glycol
PTA	Phosphotungstic acid
PSar	Polysarcosine
ROP	Ring opening polymerization
RT	Room temperature (≈ 20 °C)

1. Materials and instrumentation

All chemicals used were of commercial grade and used directly without purification.

^1H and DOSY spectra were measured on an ASCEND 400 BRUKER apparatus in $CDCl_3$ and MeOD. *J* values are given in Hz.

TEM images were recorded on JEOL TEM 1400 on carbon copper grids.

CryoTEM images were recorded on JEOL 2100.

DLS measurements were done on the DLS zetasizer pro in MilliQ water.

Osmolality was measured on Gonotec Osmaat 3000.

CD measurements were done on JASCO J-815 CD.

DSC measurements were performed on the DSC822^e with the TSO 801RO Sample Robot from Mettler Toledo.

The polydispersity was determined with Shimadzu GPC with THF as a solvent.

Fluorescence spectroscopy was measured on JASCO FP-8300ST.

The centrifuges used to spin down product were the HERMLE Z306 for falcon tubes and Centrifuge 5430 R for Eppendorfs.

CMC was determined to on NanoSight LM10.

2. Experimental

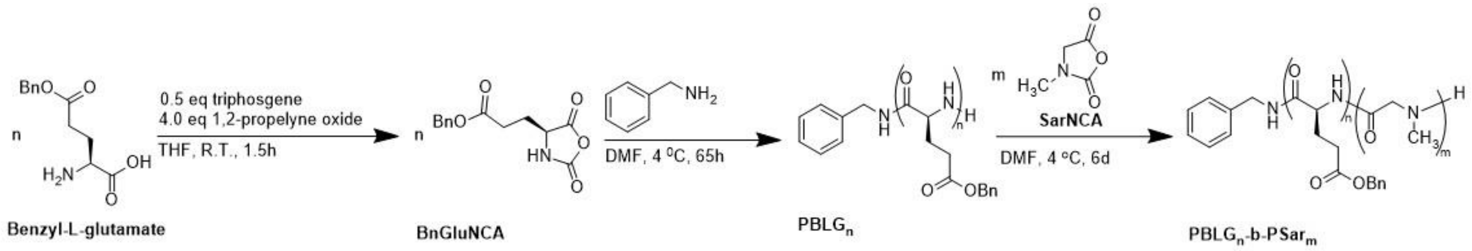

***Figure S1**: Overall scheme of PBLG$_n$-b-PSar$_m$ preparation.*

2.1. Synthesis benzyl glutamate NCA

γ-Benzyl L-glutamate (1.51 g, 6.36 mmol, 1 equiv), THF (30 mL) and 1,2-propylene oxide (4 equiv) were added to a flask under continuous stirring. Subsequently, triphosgene (1.012 mg, 3.31 mmol, 0.54 equiv) was added in one portion to the flask. γ-Benzyl L-glutamate dissolved with noticeable heat release. The mixture was stirred at room temperature for 1.5 h and subsequently cooled to 0 °C. Thereafter, ice chilly water (30 mL) was added to the mixture to quench any excess phosgene. The mixture was stirred for 3 min, to release CO_2 gas. Argon was bubbled through the mixture for a period of 15 min to quench excess phosgene. The mixture was then extracted with ethyl acetate (2 × 30 mL). The combined organic layers were extracted with brine (2 × 30 mL) and dried over anhydrous Na_2SO_4. The product was concentrated under vacuo, which was further dried on high vacuum for 16 h to obtain **BnGluNCA** as a white powder (1.60 g, 5.34 mmol, 95.2% yield). The product was stored at −20 °C.

^{1}HNMR (400 MHz, $CDCl_3$) δ 7.4-7.33 (m, 5H, Ar-H), 6.35 (s, 1H, NH), 5.14 (s, 2H, CH2), 4.39-4.35 (t, 1H, CH), 2.62-2.09 (m, 2H, CH2)

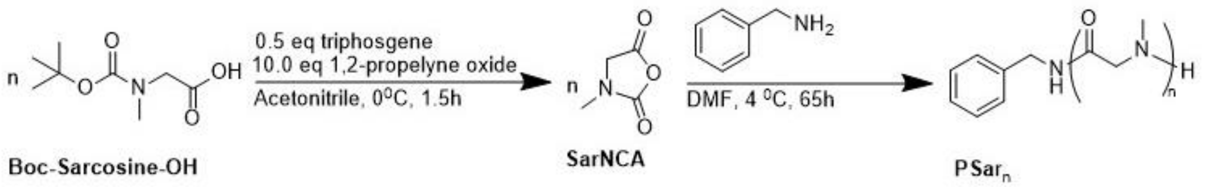

Figure S2: *Overall scheme of SarNCA synthesis and subsequent polymerisation of PSar.*

2.2. Synthesis of sarcosine NCA

Boc-Sar-OH (1.276 g, 6.74 mmol, 1 equiv) and 1,2-propylene oxide (4.7 mL, 67.1 mmol, 10.0 equiv) were dissolved in dry acetonitrile (30 mL). Finally, triphosgene (1.080 g, 3.64 mmol, 0.54 equiv) was added in one portion to the mixture. Two needles were stuck into the septum in order to facilitate in situ deprotection of Boc-Sar-OH under a constant stream of argon. The mixture was stirred while cooled on ice for 1.5 h. After completion, ice chilly water (15 mL) was added to the reaction mixture to quench any excess phosgene in the mixture and stirred for an additional 5 minutes. The resulting mixture was extracted with ethyl acetate (2 × 30 mL). The combined organic phase was subsequently washed with brine (3 × 30 mL). The organic phase was dried over anhydrous Na_2SO_4. The product was concentrated in vacuo. A transparent oil was obtained and subsequently dried for 16 h on high vacuum to obtain white crystals **SarNCA** (527.7 mg, 4.59 mmol, 68.0% yield) as a white powder. The product was stored at −20 °C.

^{1}HNMR (400 MHz, $CDCl_3$) δ 4.13 (s, 2H, CH2), 3.05 (s, 3H, CH3)

2.3. Synthesis of $PBLG_{40}$

BnGluNCA (202.7 mg, 0.77 mmol, 40 equiv) was dissolved in dry DMF (3 mL) under a continuous Ar flow. Subsequently, a solution of 0.0366 mM benzylamine in dry DMF (0.52 mL, 0.019 mmol, 1 equiv) was added to the solution. The reaction mixture was stirred for 65 h at 4 °C. Thereafter, the mixture was carefully pipetted into stirred, ice cold diethyl ether (150 mL). The white precipitates were separated through centrifugation and subsequently freeze-dried from H_2O/1,4-dioxane 50:50 to obtain **$PBLG_{40}$** (146.3 mg, 0.016 mmol, 86.7% yield) as a fluffy white powder. Đ was 1.04 as determined by GPC with THF as eluent.

^{1}HNMR (400 MHz, $CDCl_3$) δ 8.30 (bs, NH), 7.32-7.19 (m, Ar-H), 5.37 (s, 79H, Ar-CH2NH), 4.34 (bs, 76H, CH2-Ar), 3.93 (bs, 33H, CH), 2.57-2.12 (m, 154 H, CH2)

2.4. Synthesis of $PBLG_{40}$-b-$pSar_{50}$

$PBLG_{40}$ (149.9 mg, 0.0169 mmol, 1 equiv) was dissolved in dry DMF (1 mL) and cooled to 0 °C. **SarNCA** (97.2 mg, 0.884 mmol, 50 equiv) was dissolved in dry DMF under constant argon flow. Subsequently, the **$PBLG_{40}$** solution in DMF was added to the monomer solution of **SarNCA** under argon flow. The mixture was stirred at 4 °C for 6 days. Thereafter, the mixture was carefully pipetted into stirred, ice cold diethyl ether (50 mL). The white precipitates were separated by centrifugation and subsequently washed with diethyl ether (2 × 50 mL). The precipitates were freeze-dried from H_2O/1,4-dioxane 50:50 to obtain **$PBLG_{40}$-b-$PSar_{50}$** (176.5 mg, 0.0142 mmol, 84.0% yield) as a fluffy white powder. Đ was 1.07 determined by GPC with dimethylacetamide as eluent.

^{1}HNMR (400 MHz, $CDCl_3$) δ 8.30 (bs, NH), 7.29-7.23 (m, ArH), 5.02 (s, 80H, Ar-CH2NH), 4.52 (bs, 2H, CH2-Ar), 3.02 (bs, 150H, CH3), 2.56-2.09 (m, CH2)

2.5. Synthesis of pSar$_{40}$

SarNCA (134.1 mg, 1.16 mmol, 44 equiv) was dissolved in dry DMF (5 mL) under argon and subsequently cooled to 0 °C. Benzyl amine (3 µL, 0.03 mmol ,1 equiv) was dissolved in dry DMF (3 mL) and cooled to 0 °C. Thereafter, the benzyl amine solution was added to the SarNCA solution under a constant stream of Ar. The resulting mixture was stirred for 90 h at 4 °C. After completion, the solution was slowly pipetted on ice cold diethyl ether (50 mL). The white precipitates were separated by centrifugation and washed with diethyl ether (3 × 50 mL). Then, the precipitates were freeze-dried from H_2O to obtain **PSar$_{40}$** (214,7 mg, 0.073 mmol, 12.1% yield) as a slightly yellow solid. Đ was determined to be 1.04 by GPC with THF as eluent.

^{1}H NMR (400 MHz, MeOD) δ 7.38 – 7.26 (m, 5H), 4.47 – 4.02 (m, 75H), 2.98 (td, *J* = 23.4, 14.7 Hz, 143H), 1.35 (d, *J* = 3.2 Hz, 2H).

3. Self-assembly and shape transformation

3.1. Preparation of BCPs polymersomes

Typically, the BCPs were dissolved in 1 ml DMF (4 mg/mL). MQ water (2 mL) was then added using Chymex syringe pumps over a period of 4 h (flow rate 0.5 mL/h) with an 11-minute delay interval under constant stirring to obtain a concentration of 1.33 mg/mL and 66% water content. After the 4 h period, MQ water (9 mL) was added in one portion to quench the self-assembly for a total concentration of 0.33 mg/mL in 92% water content. Subsequently, the mixture was centrifuged at 10,000 rpm for 10 min and the supernatant was discarded. The pellets were resuspended in MQ water (1 mL). This process was repeated two more times to obtain the washed self-assemblies.

3.2. Shape transformation of PSar-PBLG vesicles

BCPs were dissolved in 1 mL DMF (4 mg/mL). MQ water (2 mL) was then added using Chymex syringe pumps over a period of 4 h (flow rate 0.5 mL/h) with an 11-minute delay interval under constant stirring. After the 4 h period, the sample was split into parts (1.33 mg/mL) with a 66% water content. While stirring, 2000 µg of PEG2000[1] was added to the self-assembly (1 mg) before quenching the self-assembly by adding MQ water in one portion after 1 minute to obtain 92% water content. Subsequently, the mixture was centrifuged at 10,000 rpm for 10 min and the supernatant was discarded. The pellets were resuspended in MQ water (1 mL), and this process was repeated two more times to obtain the washed self-assemblies.

[1] Or 5µL of 400mg/ml PEG2000

3.3. Heated shape transformation of BCPs

The heated shape transformation was performed through a similar method as the previously mentioned shape transformation. After the self-assembly was performed using the Chymex syringe pumps, the into four split samples were heated by being held in a water bath for 1 minute at the right temperature. After this minute, 2000 µg of PEG2000 was inserted into the sample and quenched after a range of times to obtain 92% water content. In the same fashion as described prior, the mixture was cetrifuged at 10,000 rpm for 10 min and the supernatant was discarded. The pellets were resuspended in MQ water (1 mL), and this process was repeated two more times to obtain the washed self-assemblies [Figures S23–26, S28–30, S34].

3.4. Relaxation to vesicle

After shape transformation to the disk, the membrane can be relaxed back to the polymersome shape by not quenching directly and leaving the sample at room temperature for 1 h. The sample is quenched with milliQ to obtain 92% water content. In the same fashion as described prior, the mixture was centrifuged at 10,000 rpm for 10 min and the supernatant was discarded. The pellets were resuspended in MQ water (1 mL), and this process was repeated two more times to obtain the purified self-assemblies [Figure S32].

3.5. Saccharose-induced shape transformation

Saccharose and the homopolymer of sarcosine were used in a similar fashion to PEG2000 to induce a shape transformation. The saccharose was added to the split sample (1 mg) and quenched with water after 1 minute to obtain 92% water content.

The work up to obtain the washed self-assemblies was done in the same fashion as described prior. Figure S33

3.6. PSar homopolymer-induced shape transformation

The (heated) shape transformation of PSar and the polymersome formation was performed in the same manner as the earlier described method for BCP. Figure S31

3.7. Heated dialysis

After the self-assembly was performed using the Chymex syringe pumps, the solution was transferred to a dialysis bag. The bag was added to a water bath with a temperature of 70 °C to perform the dialysis. Subsequently, the bag was transferred to a new water bath at 70 °C after 5 minutes, 10 minutes, 15 minutes, and finally after 20 minutes. Then, the dialysis bag was transferred to a room temperature water bath and stirred for an additional hour. Finally, the mixture was centrifuged at 10,000 rpm for 10 min and the supernatant was discarded. The pellets were resuspended in MQ water (1 mL); this process was repeated two more times to obtain the washed self-assemblies. The vesicles were analyzed by cryo-TEM (Figure S27).

3.7. Osmometer

Osmolality is a measurement of the total number of solutes in a liquid solution expressed in osmoles of solute particles per kilogram of solvent.[1] This was done to give an indication of the osmotic strength of each of these solutions. The osmolality of the PEG_{44}, $PSar_{40}$, and saccharose solutions was measured at a concentration of 400 mg/mL (Table S1). Of each solution 50 µL was transferred to a 0.5 mL Eppendorf tube which was inserted in the osmometer.

Table S1: *Osmolality values of various samples.*

**Sample name**	**Osmolality (mOsm/kg)**
PEG400	2243
40PSar400	1129
20PSar400	1217
Sacharose400	1649

4. Dynamic light scattering

Table S2: *Average sizes of self-assembled structures of $PBLG_{40}$-b-$PSar_{50}$ with different preparation methods. Experiments were performed in triplo on DLS zetasizer pro with general purpose analysis mode.*

Sample name	Average size (nm)
Polymersome	350.8
Stomatocyte, quenched after 5 s of PEG addition [a]	460.2
Stomatocyte, quenched after 30 s of PEG addition [a]	480.9
Stomatocyte, quenched after 1 min of PEG addition [a]	474.4
Stomatocyte, quenched after 2 min of PEG addition [a]	519.6
Dialysis [a]	469.2
Polymersome, freeze dried	361.3

[a] The shape transformations were performed at 70 ºC

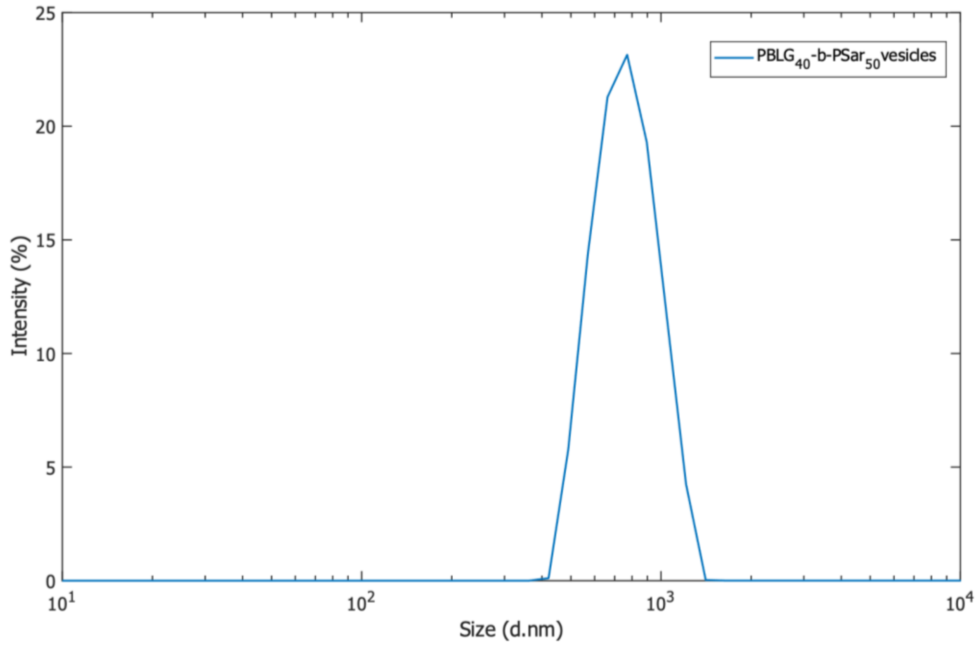

Figure S3: *DLS polymersome vesicles peak.*

5. Circular dichroism measurements

When the wavelength reaches 185 nm, the CD measurement is no longer accurate.

The parameters for these measurements include an accumulation of five measurements with a scanning speed of 200 nm/min and a digital integration time (D.I.T.) of 0.5 seconds.

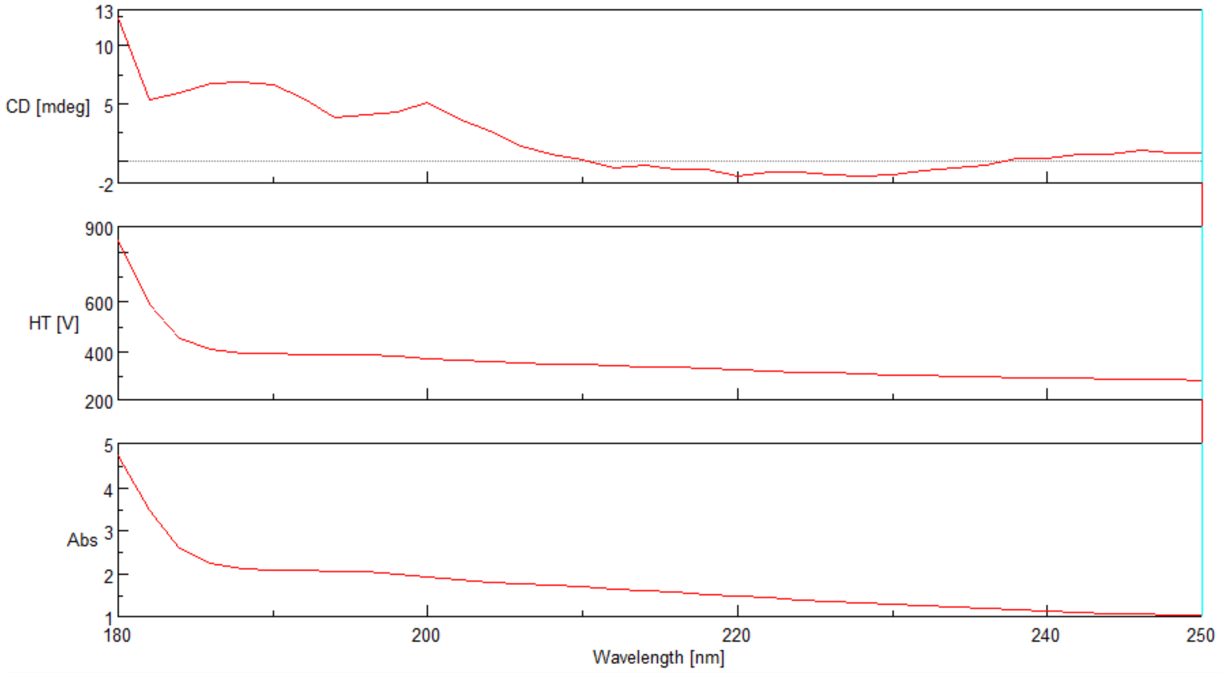

Figure S4: *CD measurement of PBLG$_{40}$-b-PSar$_{50}$ polymersomes of vesicles assembled from DMF.*

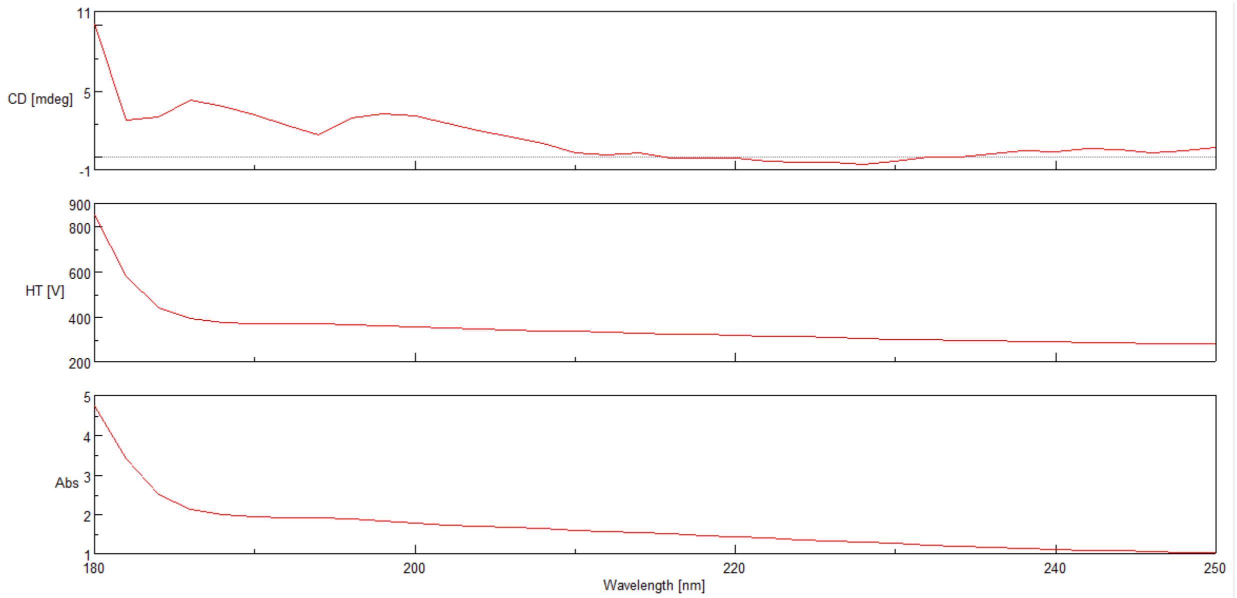

Figure S5: *CD measurement of PBLG$_{40}$-b-PSar$_{50}$ vesicles assembled from DMF after shape transformation at 70 ºC, quenched after 30s.*

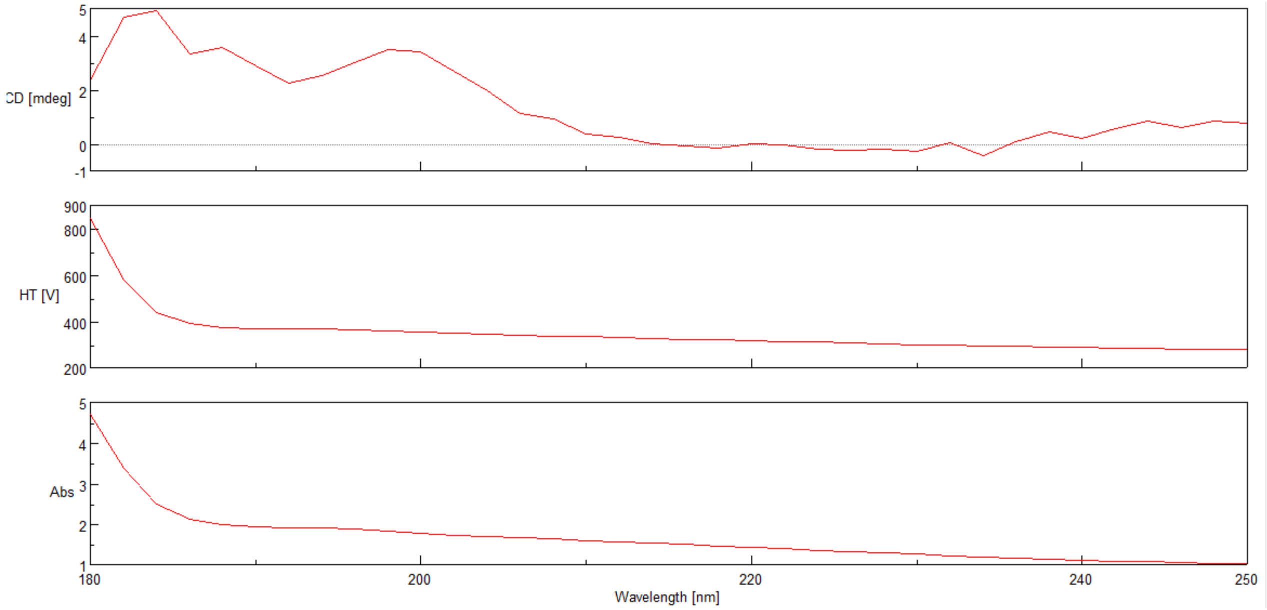

Figure S6*: CD measurement of PBLG$_{40}$-b-PSar$_{50}$ vesicles assembled from DMF after shape transformation at 70 ºC, quenched after 1 min.*

6. Spectra of NMR spectroscopy, GPC and CD

6.1. 400 MHz NMR

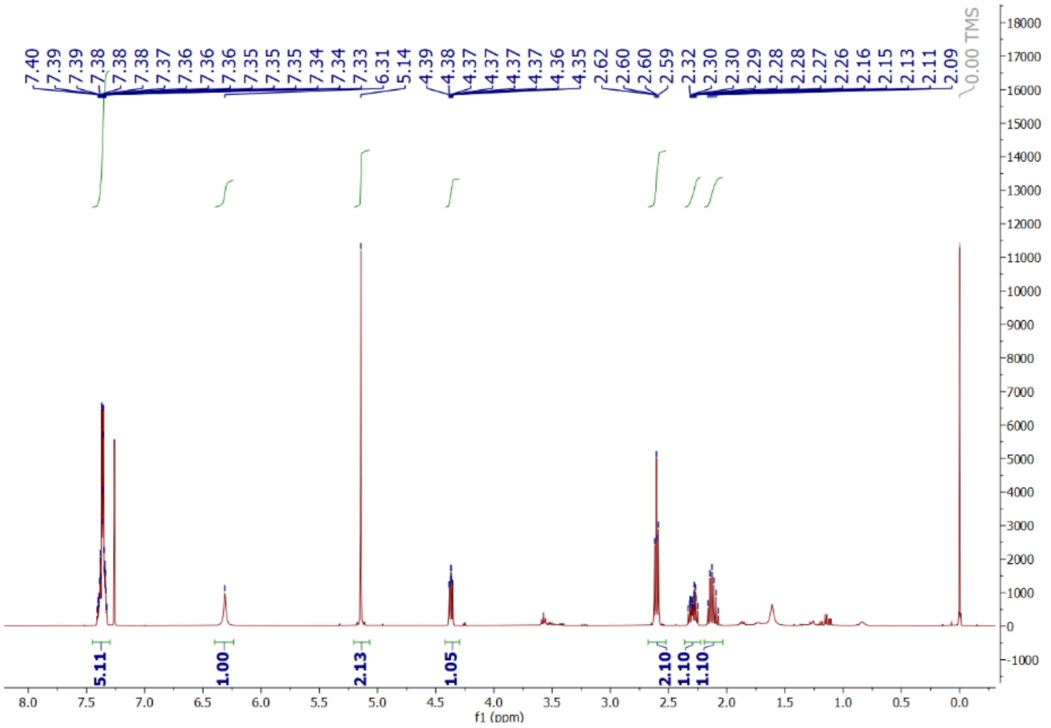

Figure S7: *¹H NMR spectrum of BnGlu NCA.*

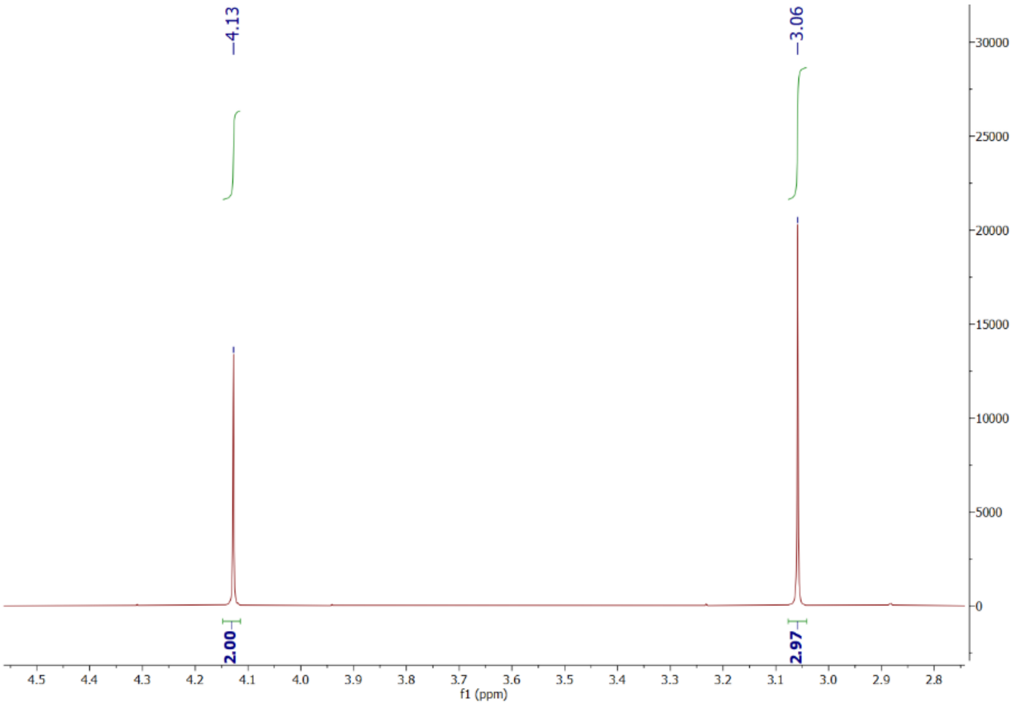

Figure S8: *¹H NMR spectrum of Sar NCA.*

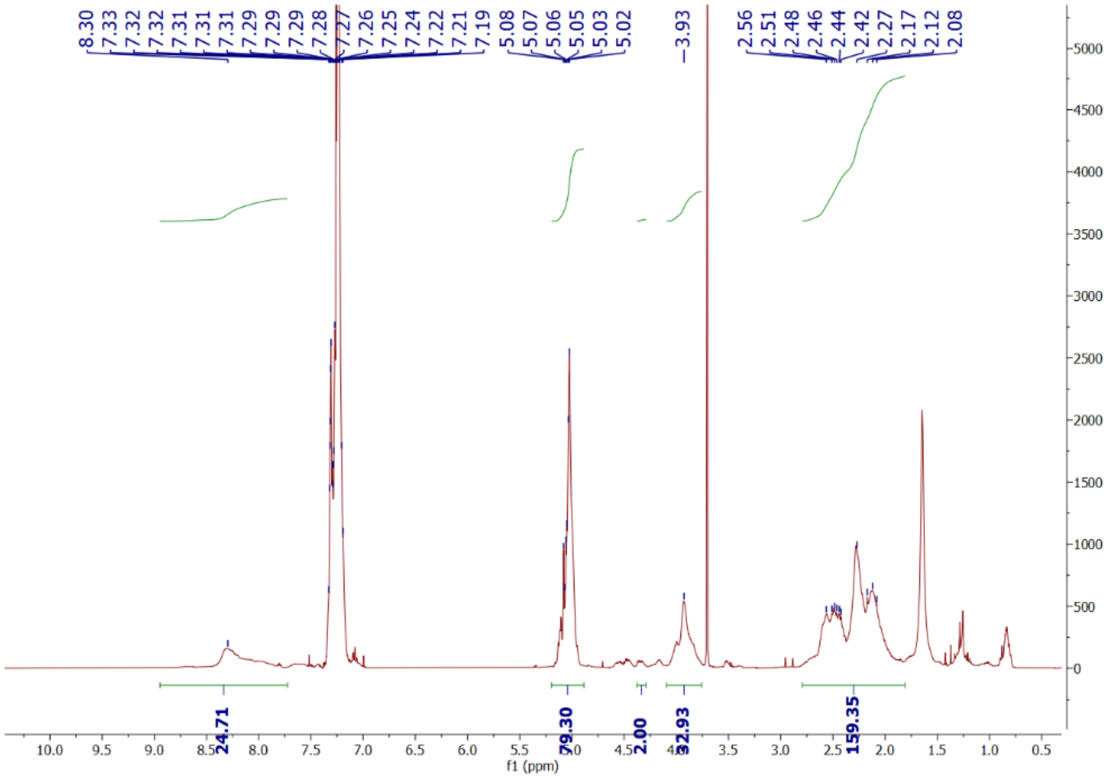

Figure S9: *^{1}H NMR spectrum of PBLG$_{40}$.*

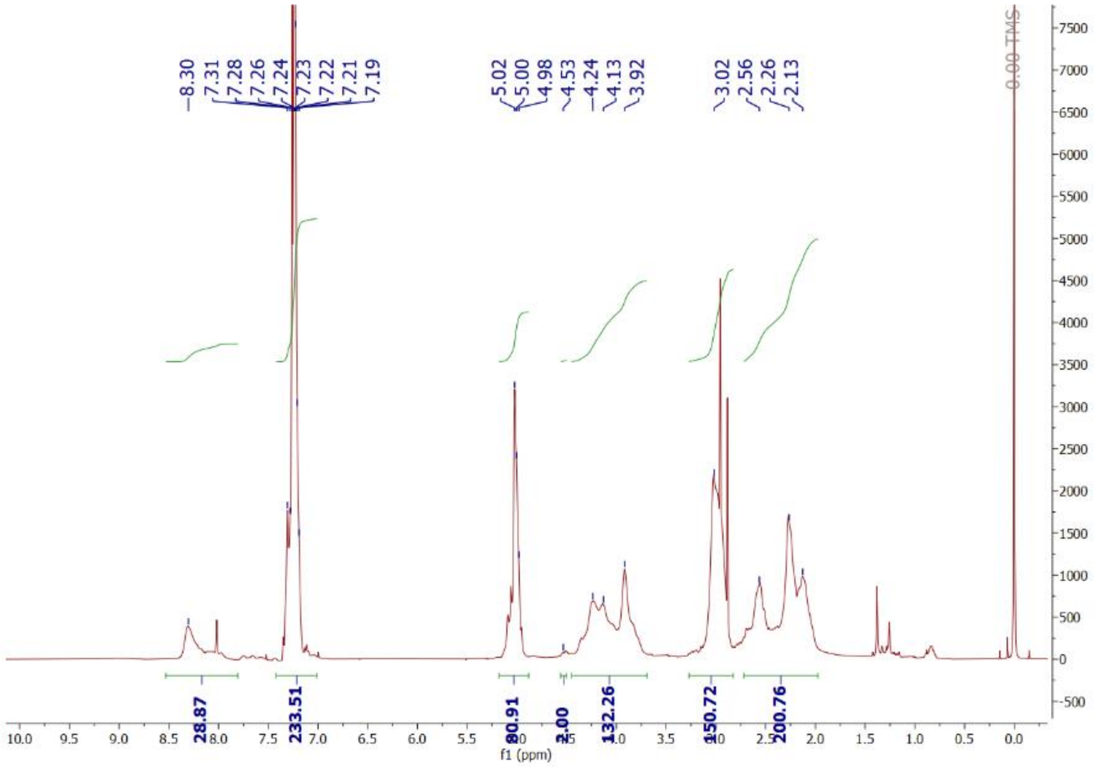

Figure S10: *^{1}H NMR spectrum of PBLG$_{40}$-b-PSar$_{50}$.*

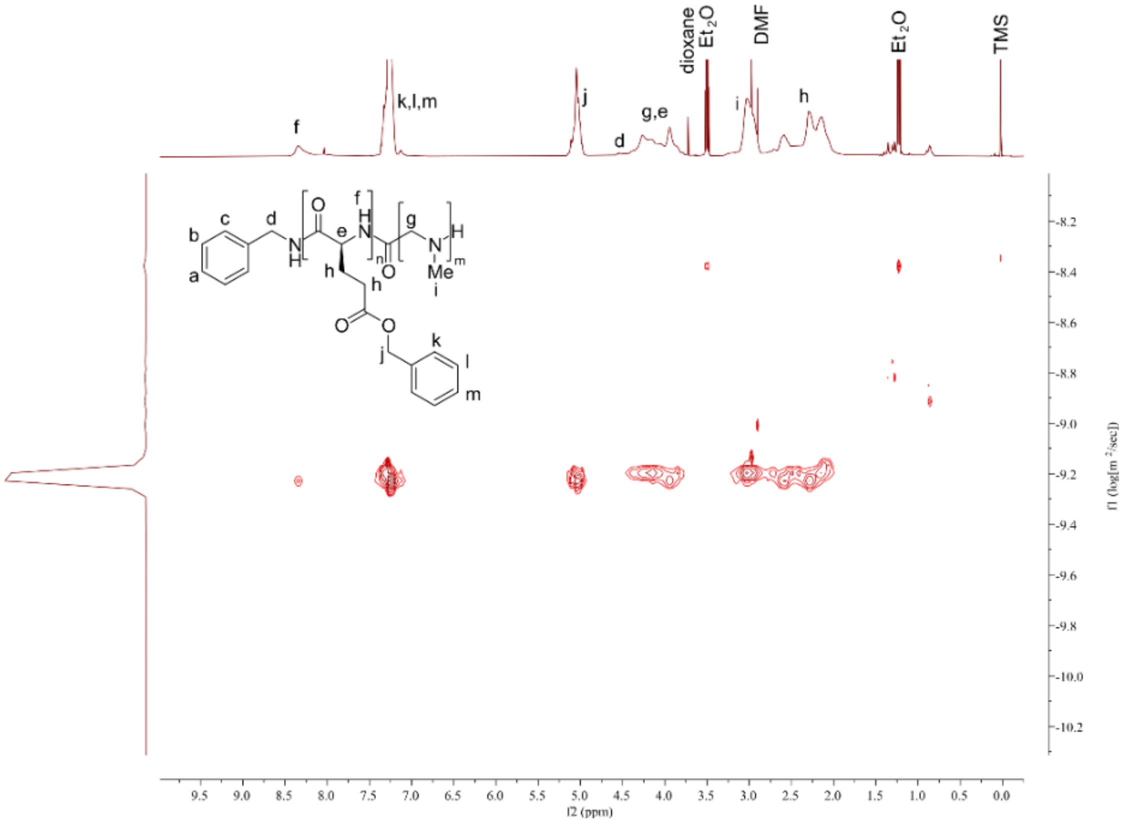

Figure S11: *DOSY spectrum of PBLG$_{40}$-b-PSar$_{50}$.*

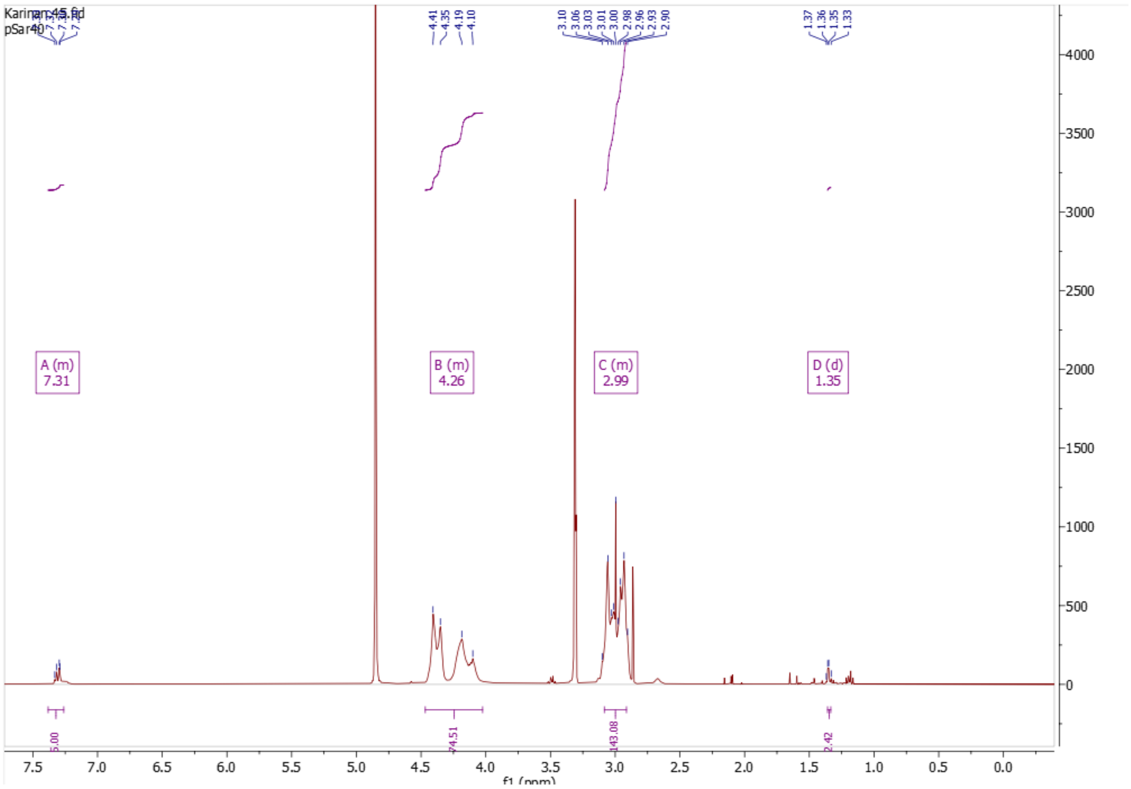

Figure S12: *^{1}H NMR spectrum of PSar$_{40}$.*

6.2. NMR under heating

The self-assembly process from S3.1. was followed but instead of milliQ D_2O was used. In the end the sample was concentrated to 4 mg of vesicles in 0.5 mL D_2O. An ^{1}H NMR spectrum was measured at intervals of 10 degrees starting at 30 °C and ending at 70 °C. In Figure S13 and S14 a clear decrease in signal of the PSar CH_3 can be observed as well as clear peak broadening. Upon heating the chemical shift went from 3.3 to 3.8 ppm, for the purpose of comparing the peaks they were all set at 3.3 ppm.

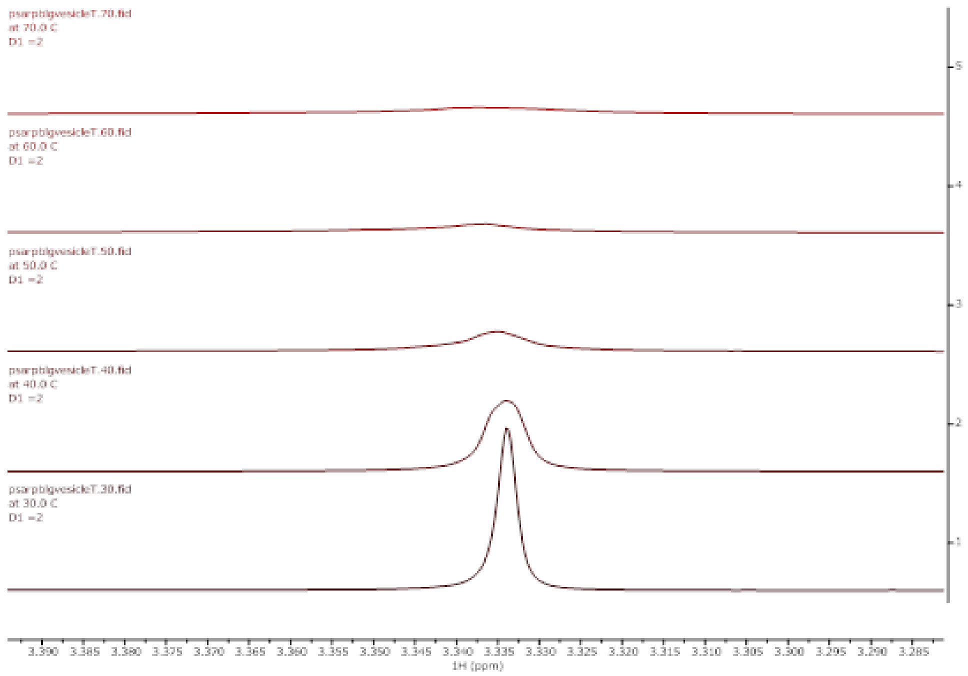

Figure S13: *The signal of PSar in HNMR tracked over 30-70°C.*

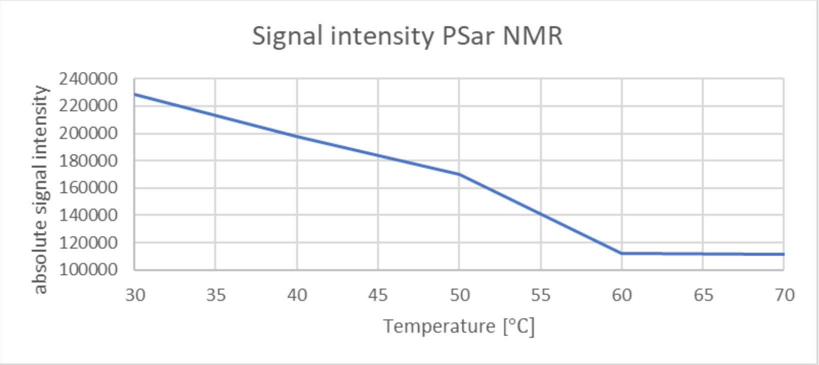

Figure S14: *Signal intensity of PSar in PSar-PBLG vesicles tracked over time.*

6.3. Gel permeation chromatography

The polydispersity of the polymers was determined in THF.

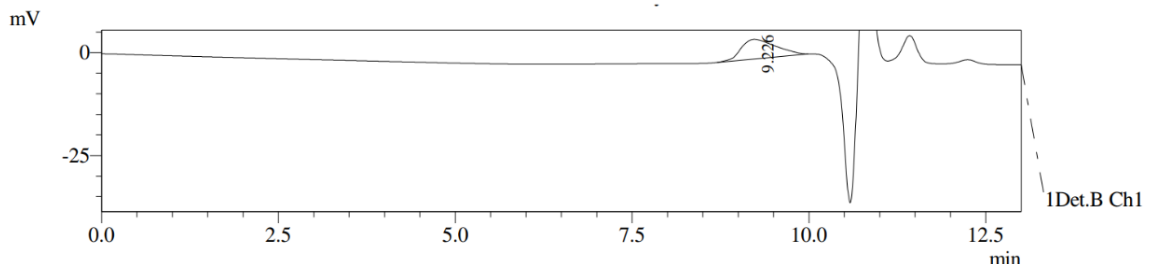

Figure S15: *GPC trace of PBLG$_{40}$. The PDI was determined to be 1.04.*

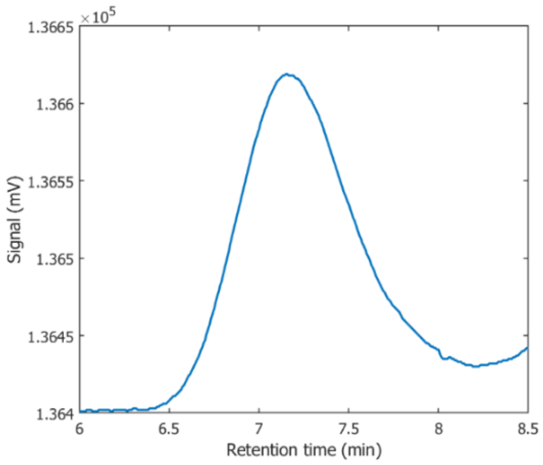

***Figure S16**: GPC trace of PBLG$_{40}$-b-pSar$_{50}$. The PDI was determined to be 1.07.*

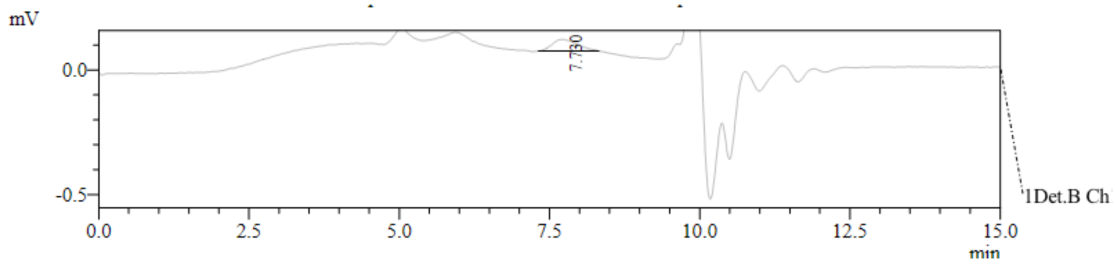

Figure S17: *GPC trace PSar$_{40}$. The PDI was determined to be 1.04.*

6.4. Differential scanning calorimetry

The thermograph for PSar$_{50}$-PBLG$_{40}$ was measured at a rate of 10 °C/min. The first heating cycle was from 20 °C to 120 °C and then back to 20 °C. The second and third heating cycle were both from 20 °C to 180 °C and back to 20 °C. The resulting thermograph can be seen in Figure S18 with an indication for the peak of the dehydration at 72 °C and glass transition at 105 °C.

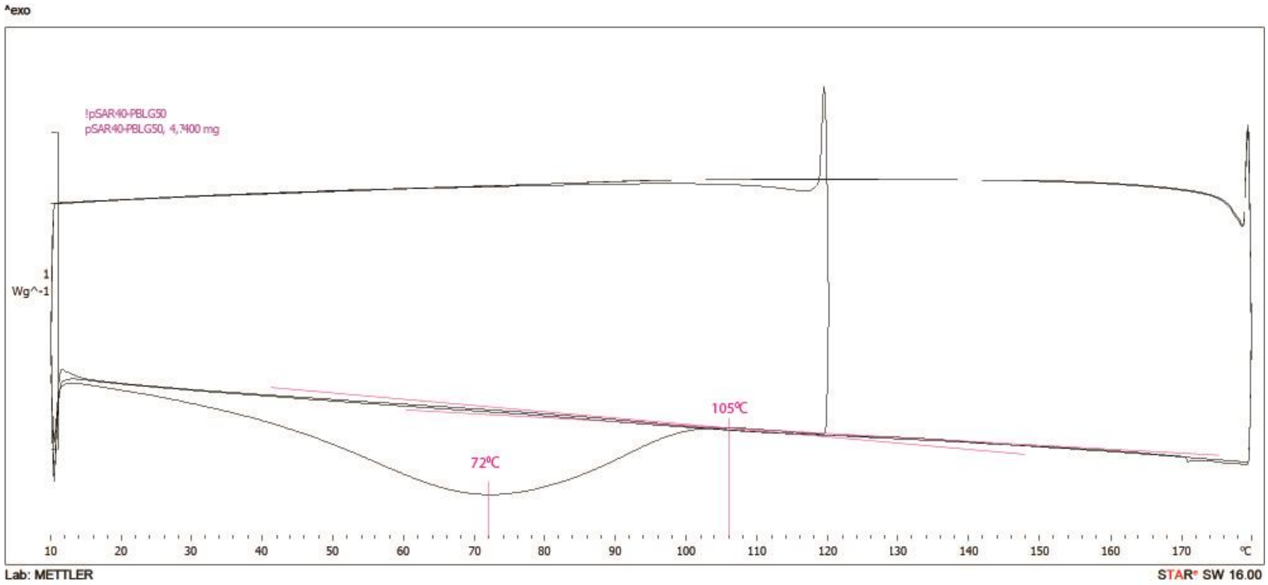

***Figure S18**: Thermogram of PBLG$_{40}$-PSar$_{50}$ with the peak for dehydration at 72 °C and the glass transition temperature at 105 °C.*

7. TEM and cryo-TEM images

7.1. TEM sample preparation and analysis

The self-assembled suspension in MQ water (0.5 mg/mL), 5 µL was pipetted onto the carbon-coated TEM grid. The suspension was left to sit for 1 min before removing the liquid using a filter paper. Subsequently, 1% phosphotungstic acid (PTA) staining (3 µL) was pipetted onto the TEM grid and left to sit for 1 min before being removed by filter paper. The TEM grid was then left to dry for at least 4 h before analysis. The TEM samples were analyzed on a JEOL TEM-1400Flash operated at 80 kV equipped with a Matataki Flash sCMOS camera.

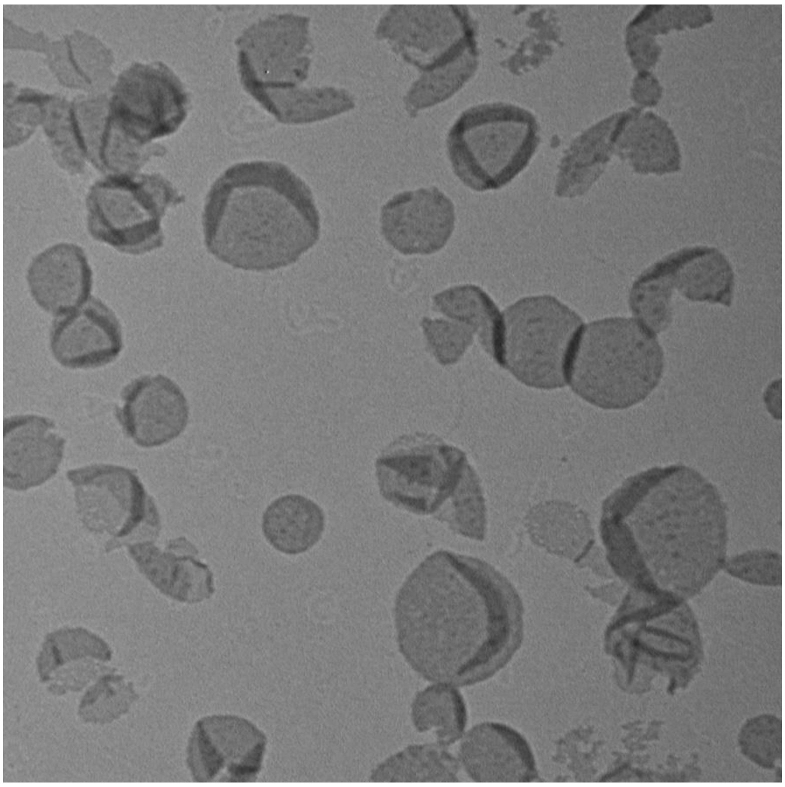

Figure S19: *TEM image of PBLG$_{40}$-b-PSar$_{50}$ polymersome. PTA staining was used.*

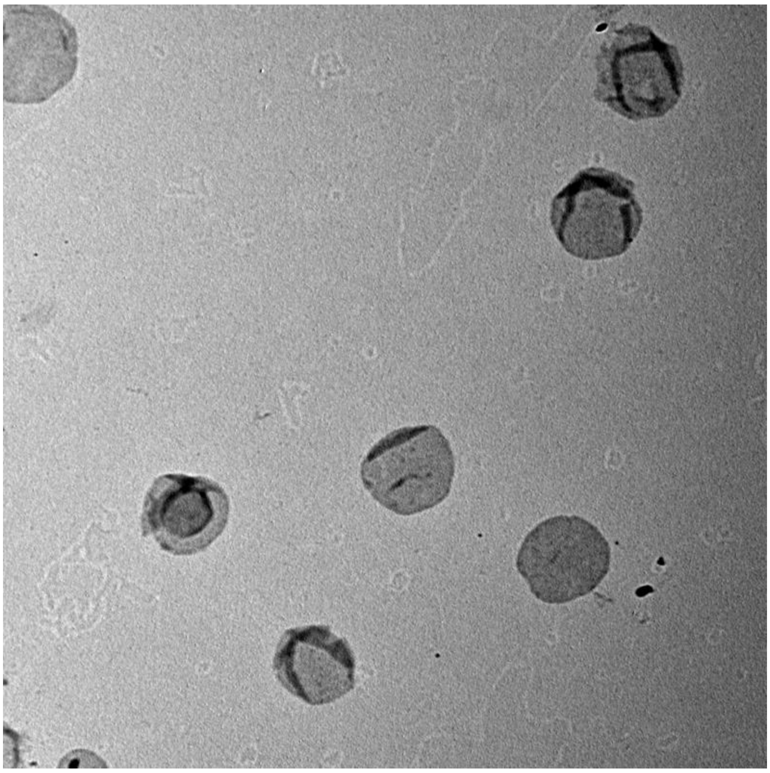

***Figure S20**: TEM image of shape transformation of PBLG$_{40}$-b-PSar$_{50}$ at 70ºC, quenched after 1 minute. PTA staining was used.*

TEM HFIP Self-assembly

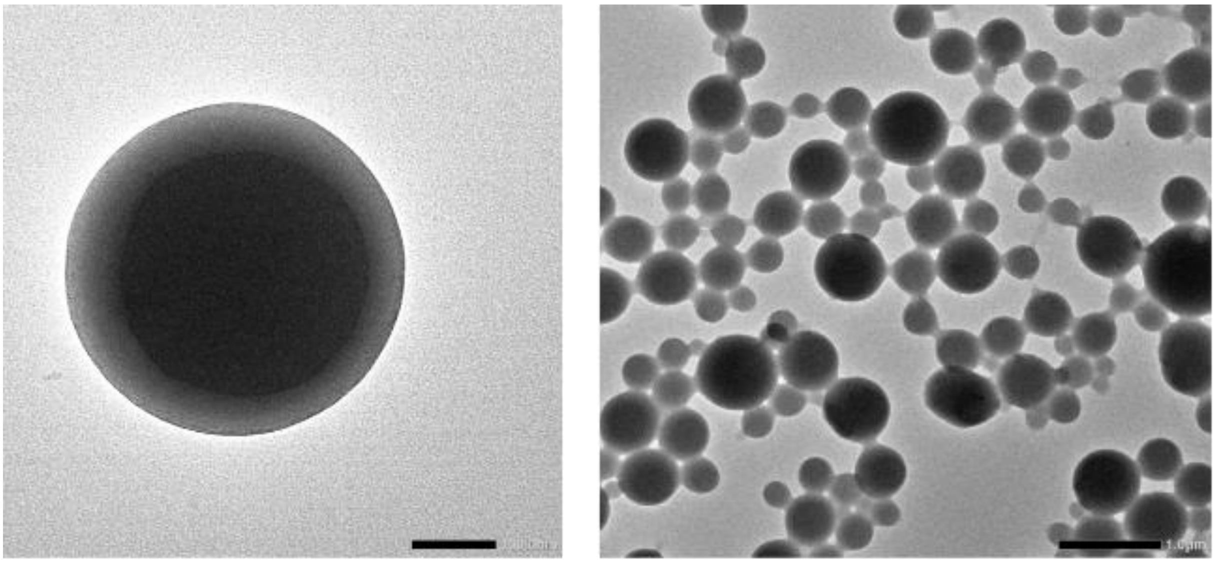

***Figure S21**: TEM images of vesicles assembled in HFIP; the vesicles have a much darker appearance compared to vesicles assembled from DMF.*

7.2. Cryo-TEM sample preparation and analysis

The carbon-coated holey film supported by a copper grid (R2/2 Cu200) was first treated with plasma at 15 kV for 40 seconds. The grid was then placed in a controlled climate chamber at 20 °C with a relative humidity near saturation. Five microliters of the vesicle sample (approximately 5 mg/mL) were deposited onto the grid, which was subsequently rapidly frozen in liquid ethane at −180 °C and then transferred to liquid nitrogen at −196 °C. The specimen was then transferred to a JEOL TEM 2100 using a cryo holder. The acceleration voltage was set to 200 kV.

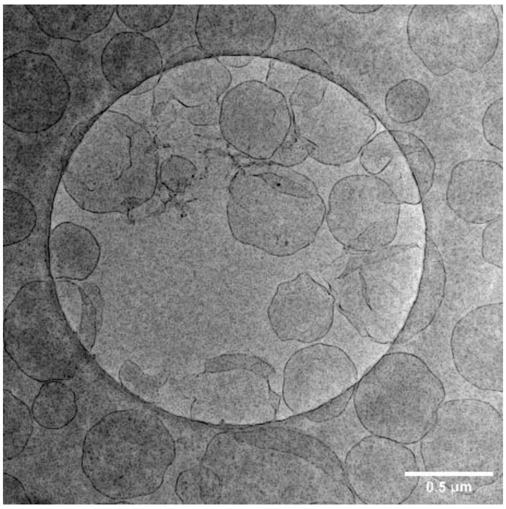

***Figure S22**: Cryo TEM image of $PBLG_{40}$-b-$PSar_{50}$ vesicles assembled from DMF.*

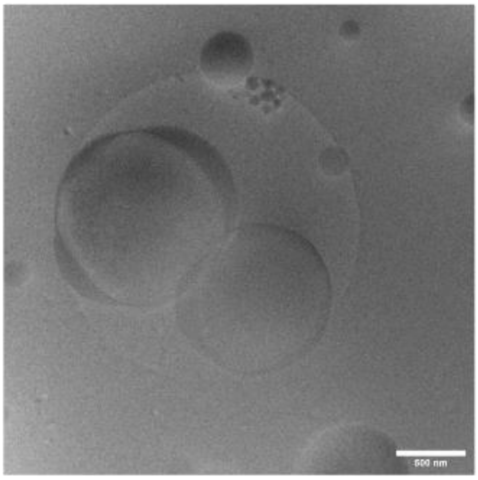

Figure S23: Cryo TEM image of $PBLG_{40}$-b-$PSar_{50}$ vesicles assembled from HFIP.

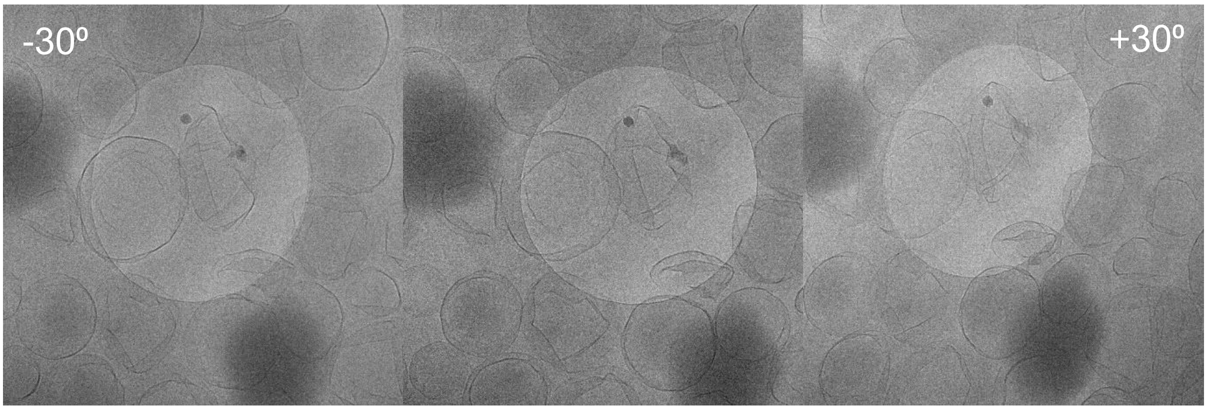

***Figure S24**: Cryo TEM image of PBLG$_{40}$-b-PSar$_{50}$ vesicles after 1 min of PEG addition at room temperature, with the left and right panel being -30° and +30° respectively.*

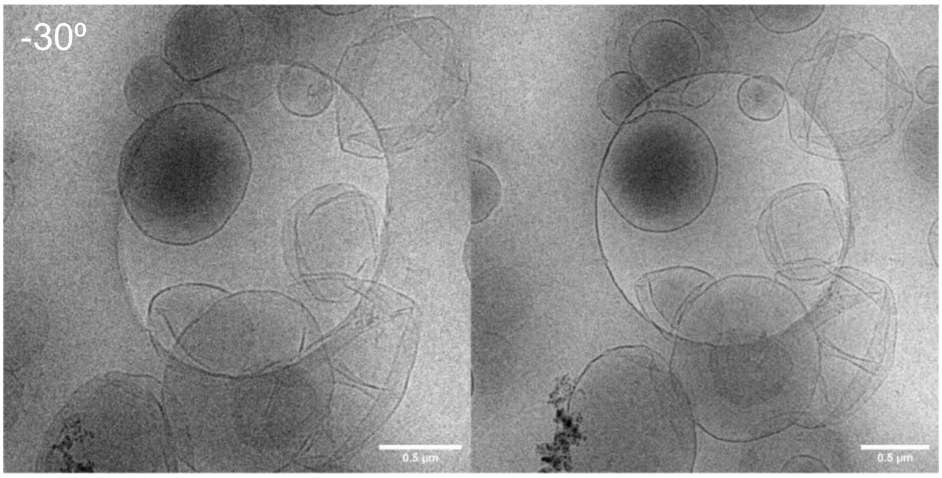

***Figure S25**: Cryo TEM image of PBLG$_{40}$-b-PSar$_{50}$ vesicles after 1 min of PEG addition at 60°C, with the left panel being -30°.*

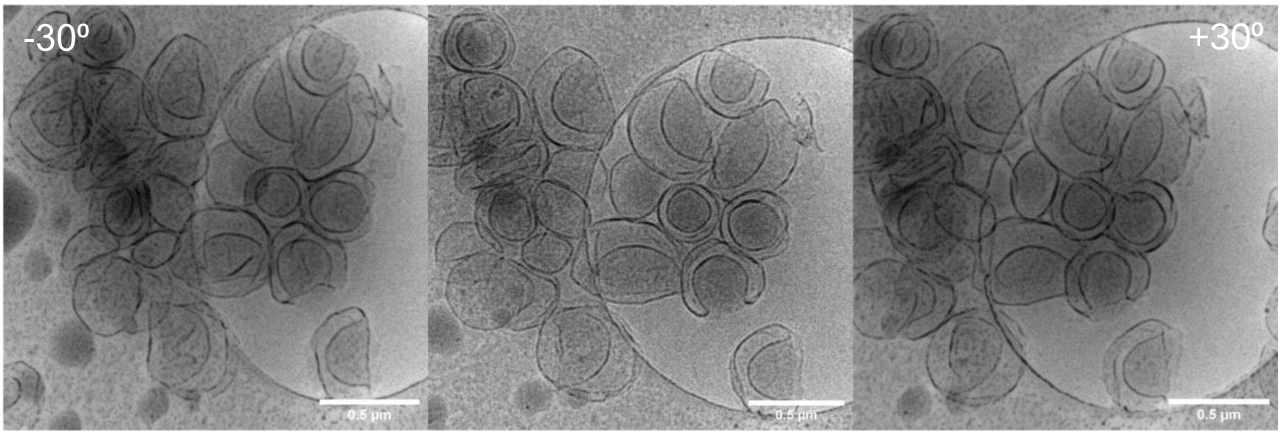

***Figure S26**: Cryo TEM image of PBLG$_{40}$-b-PSar$_{50}$ vesicles after 1 min of PEG addition at 70°C, with the left and right panel being -30° and +30° respectively.*

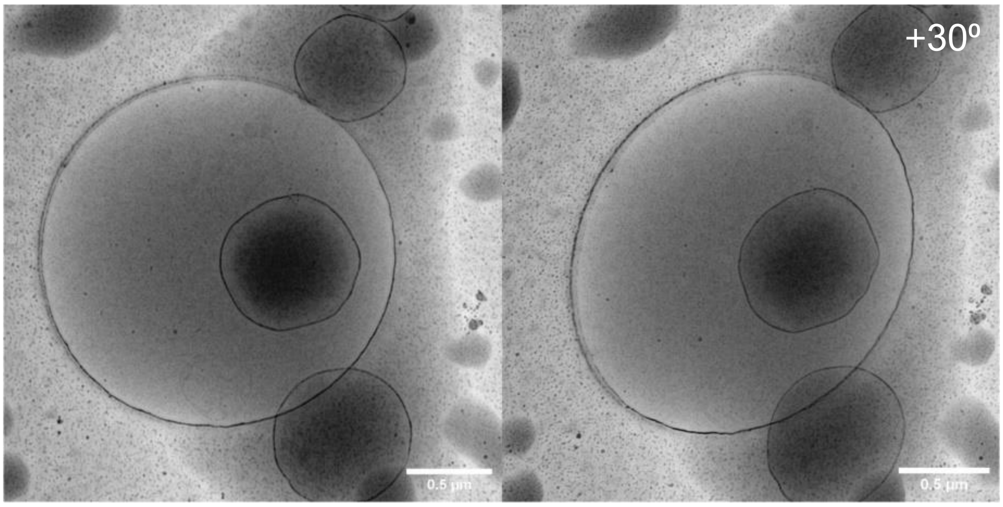

Figure S27: *Cryo TEM image of PBLG$_{40}$-b-PSar$_{50}$ vesicles after 1 min of PEG addition at 75°C, with the right panel +30°.*

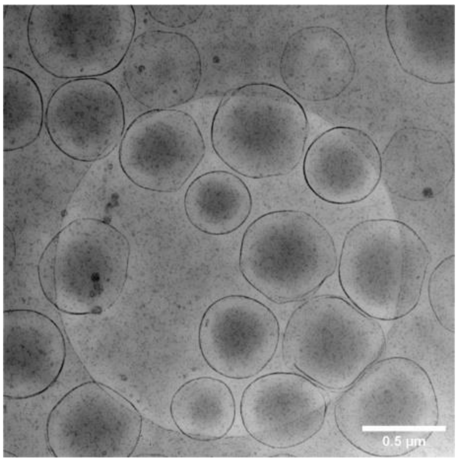

Figure S28: *Cryo TEM image of PBLG$_{40}$-b-PSar$_{50}$ vesicles after 1 min of PEG addition at 80°C.*

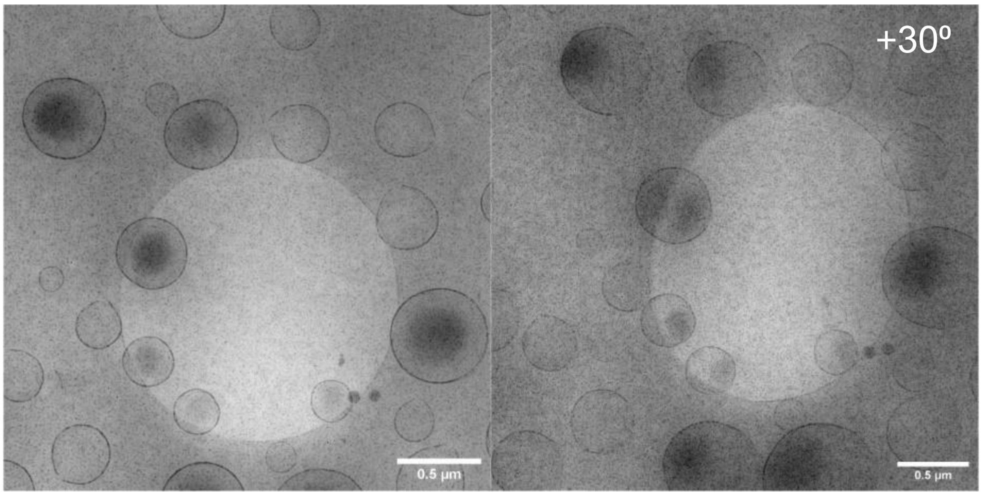

Figure S29: *Cryo TEM image of PBLG$_{40}$-b-PSar$_{50}$ vesicles after dialyses at 70°C with the right panel at +30°.*

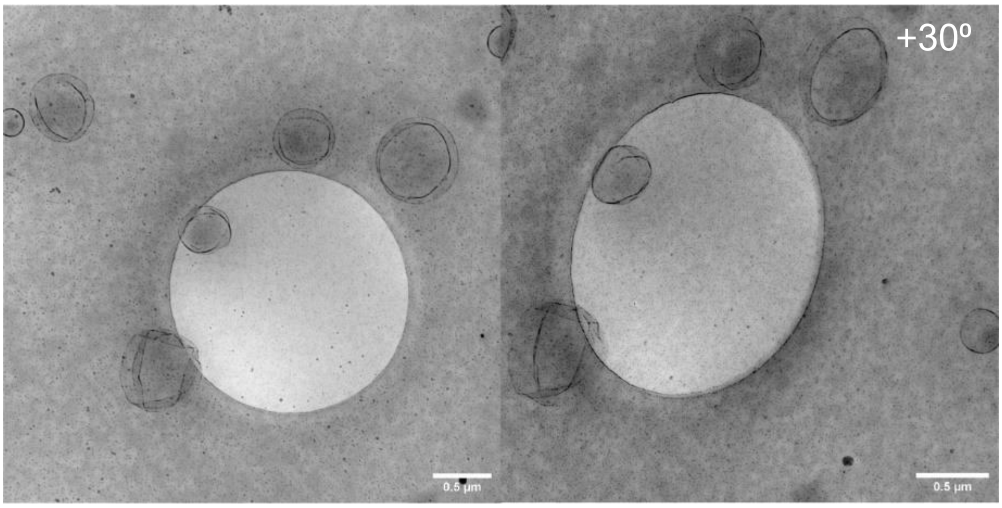

***Figure S30**: Cryo TEM image of PBLG$_{40}$-b-PSar$_{50}$ vesicles after 2 min of PEG addition at 70°C, with the right panel at +30°.*

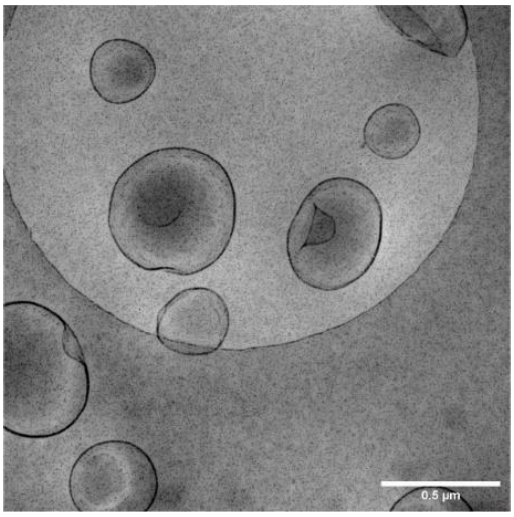

***Figure S31**: Cryo TEM image of PBLG$_{40}$-b-PSar$_{50}$ vesicles after 5 sec of PEG addition at 70°C.*

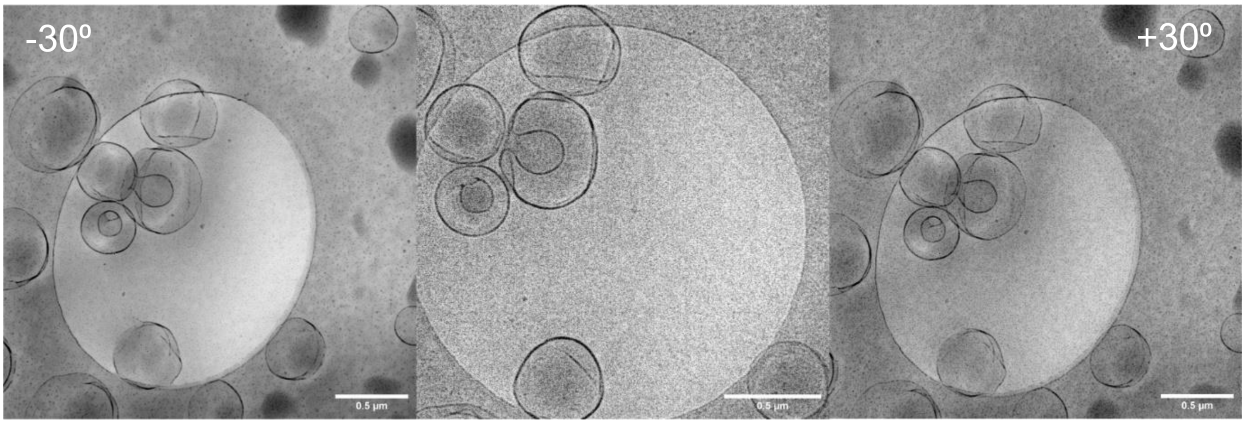

Figure S32: *Cryo TEM image of PBLG$_{40}$-b-PSar$_{50}$ vesicles after 30 sec of PEG addition at 70°C, with the left and right panel being -30° and +30° respectively.*

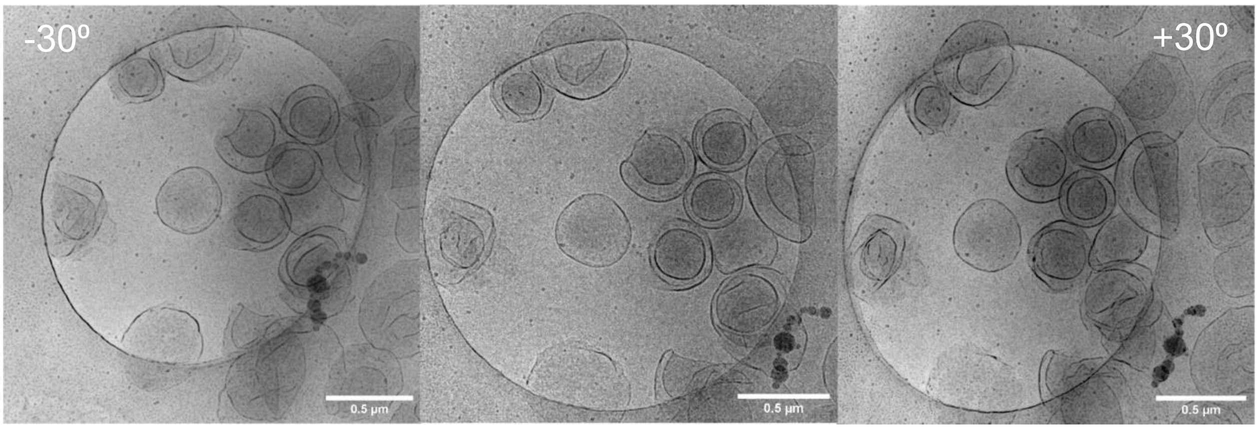

Figure S33: *Cryo TEM image of PBLG$_{40}$-b-PSar$_{50}$ vesicles after 1 min of PSar addition at 70°C, with the left and right panel being -30° and +30° respectively.*

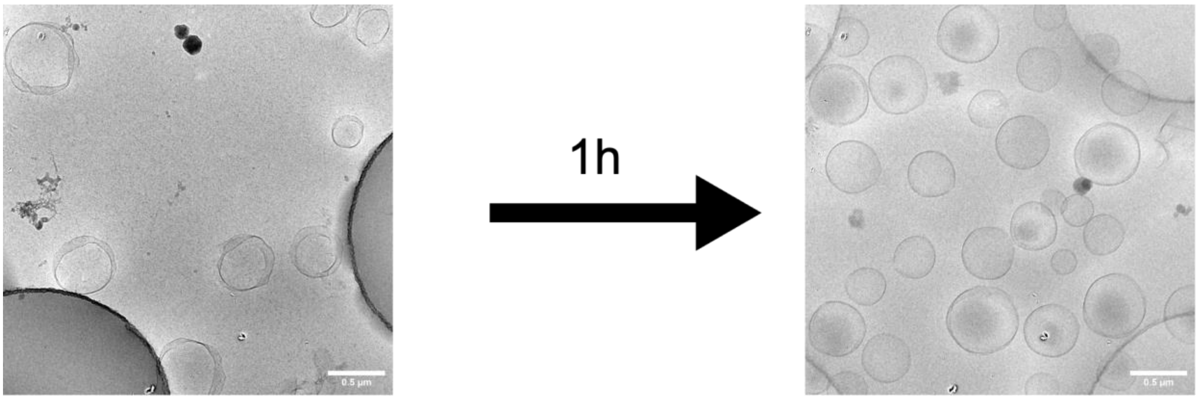

Figure S34: *Cryo TEM image of PBLG$_{40}$-b-PSar$_{50}$ vesicles after 2 min of PEG addition at 70°C and after 1 h of relaxation at room temperature.*

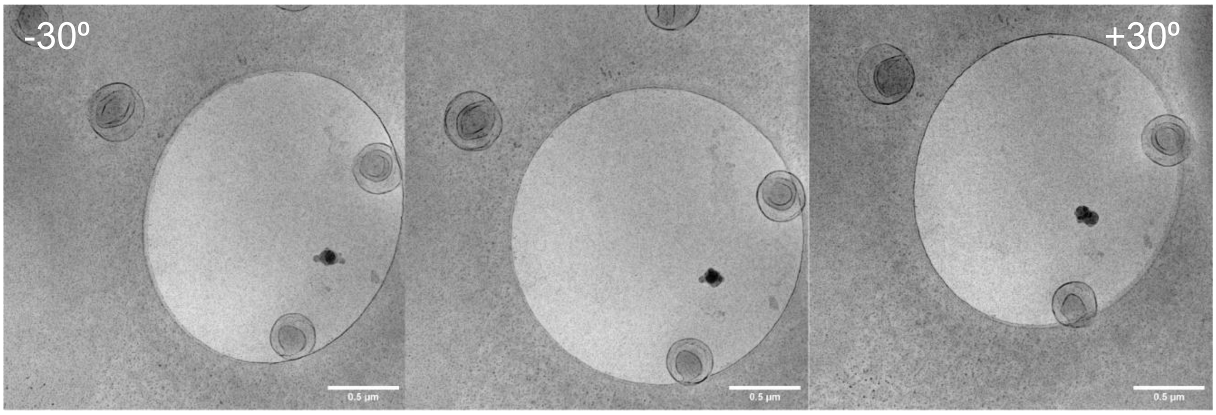

***Figure S35**: Cryo TEM image of $PBLG_{40}$-b-$PSar_{50}$ vesicles after 1 min of saccharose addition at 70°C with the left and right panel being -30° and +30° respectively.*

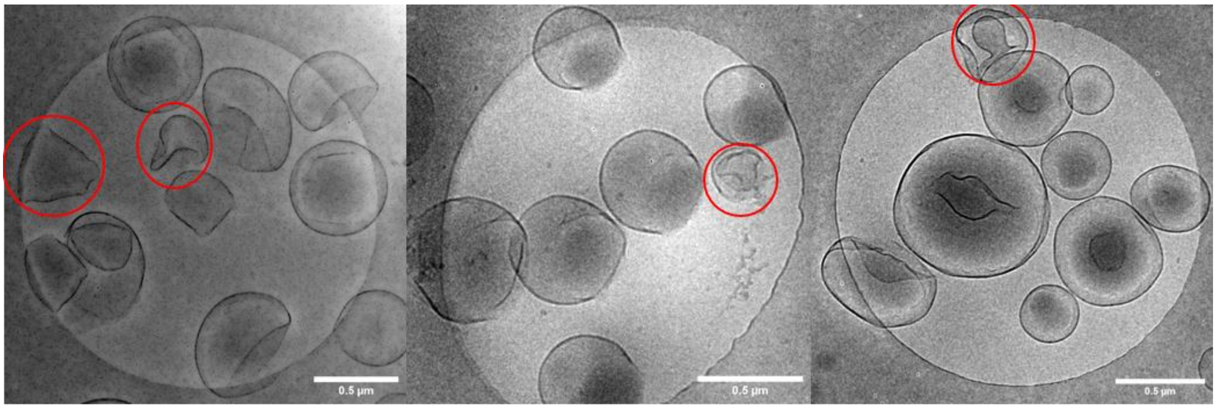

***Figure S36**: Some structures found between 5 and 30 seconds of PEG addition can have an anomalous shape. These shapes can also be found as metastable shapes of red blood cells[2].*

8. Behaviour of the membrane study

8.1. Temperature interval of PBLG$_{40}$-b-pSar$_{50}$ polymersome on DLS

The polymersomes were dissolved in MilliQ and the size was measured continuously at different temperatures in triplo general purpose data processing. The data points are spaced out with 10 °C interval.

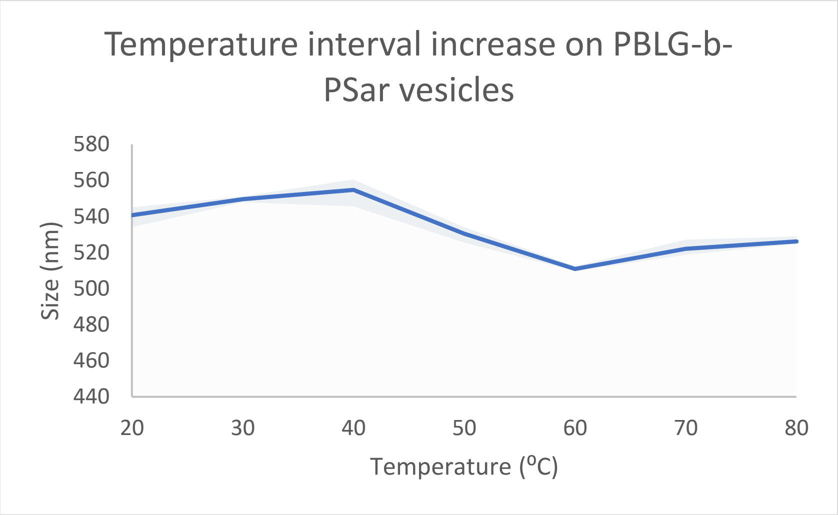

Figure S37: *DLS temperature interval experiment on PBLG$_{40}$-b-PSar$_{50}$ vesicles with increasing temperature from 20ºC to 80ºC shows the average size of the vesicles. Experiments were done in triplo.*

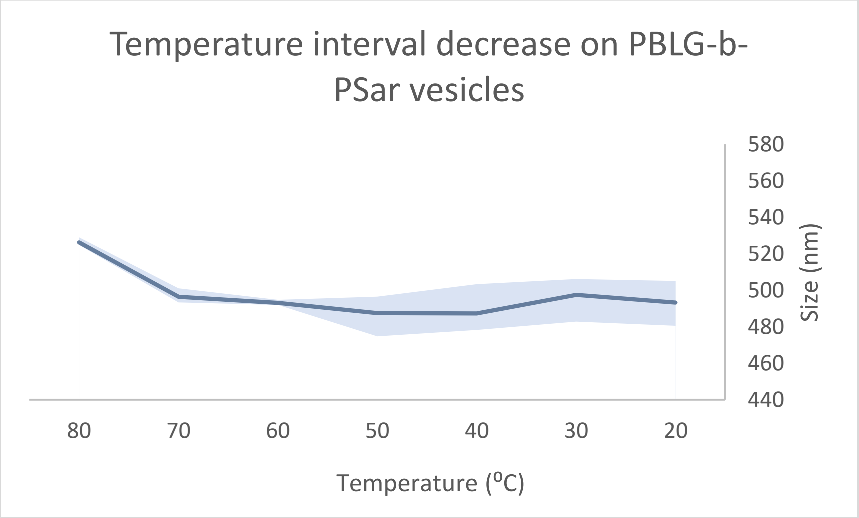

Figure S38: *DLS temperature interval experiment on PBLG$_{40}$-b-PSar$_{50}$ vesicles with decreasing temperature from 80ºC to 20ºC shows the average size of the vesicles. Experiments were done in triplo.*

Table S3: *Temperature interval experiment on DLS with polymersome of PBLG$_{40}$-b-PSar$_{50}$ data points*

**Temperature (^{0}C)**	**Average size (nm)**
20	540.7
30	549.5
40	554.7
50	530.3
60	510.9
70	522.0
80	521.5
70	496.4
60	493.2
50	487.4
40	479.3
30	497.4
20	493.2

8.2. Temperature interval of $PBLG_{40}$-b-$pSar_{50}$ polymersome on CD

A temperature dependent CD was taken to see if any changes in the secondary structure of the hydrophobic part of the polymer could be observed. The changes observed do not seem to be significant over the time range from 20 °C to 80 °C (Figure S39).00

Parameters for the measurement of these vesicles include an accumulation of five measurements with a scanning speed of 200 nm/min and a D.I.T. of 0.5 seconds. For the temperature interval, a 5.0 °C data pitch was used with a 2 °C temperature gradient with a delay time of 20 seconds.

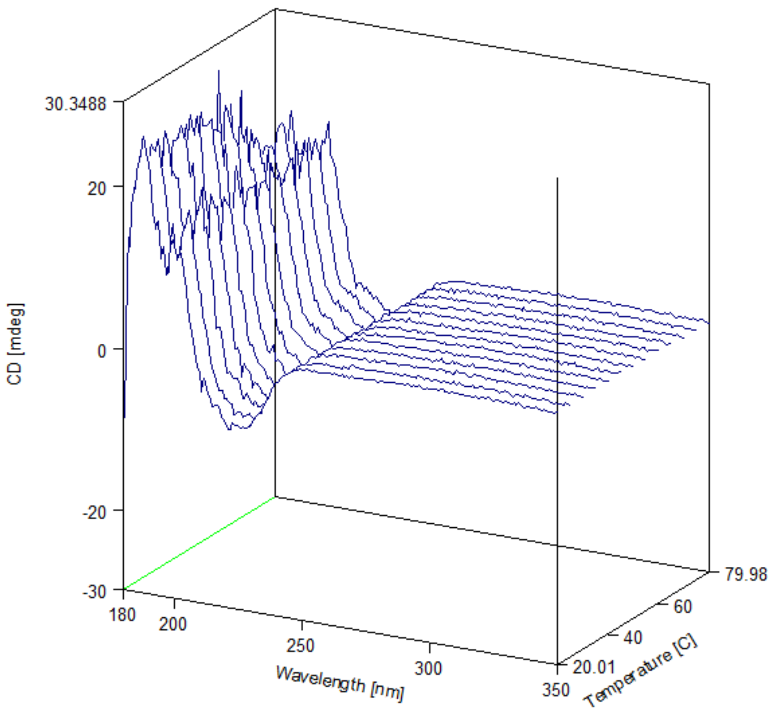

Figure S39: *Temperature interval measurement of PLBG$_{40}$-b-PSar$_{50}$ on CD*

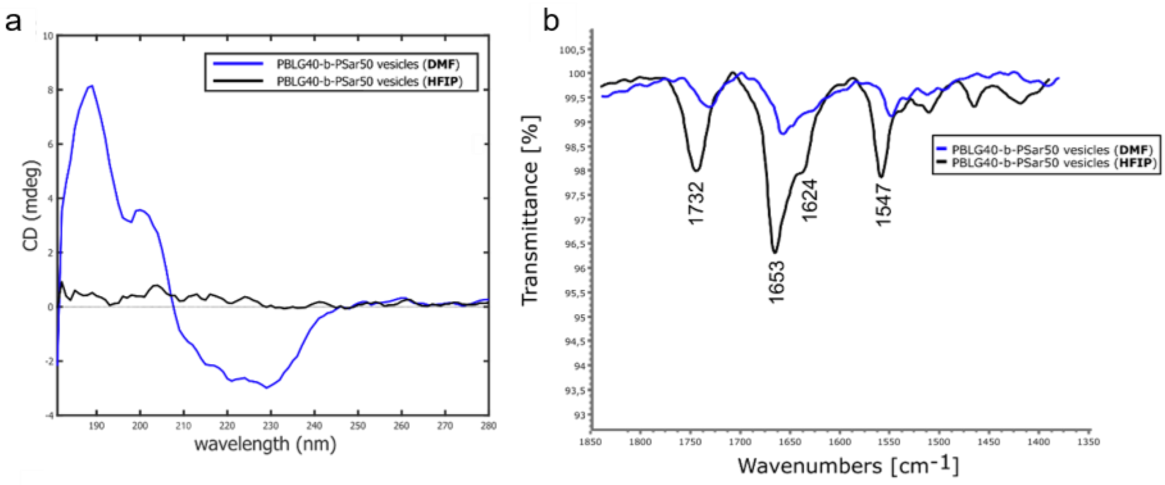

Figure S40*: a.Vesicles assembled from DMF in blue clearly showing the characteristic peak for alpha helecis and HFIP black having no clear signal. b. The IR spectra of vesicles assembled from DMF in blue with characteristic peaks for alpha helices and of vesicles assembled in HFIP in black now showing a more intents signal for the alpha helices.*

8.3. Pyrene fluorescence temperature dependence

10 mM PSar-PBLG vesicles were dissolved in MilliQ with the addition of 0.05 mM pyrene-PEG-OH [3]. The sample was excited at 339 nm. The parameters include an accumulation of three measurements with a scanning speed of 200 nm/min and a D.I.T. of 0.5 seconds. For the temperature interval, a 5.0 °C data pitch was used with a 2 °C temperature gradient with a delay time of 20 seconds. In Figure S41 the fluorescent spectra of pyrene in presence of the PSar-PBLG vesicles at 20 °C and 80 °C. The ratio of peakI at 376.2 and peakIII at 387.6 at the different temperatures can be used to determine when the hydrophobicity of the membrane changes as seen in Figure 3b.

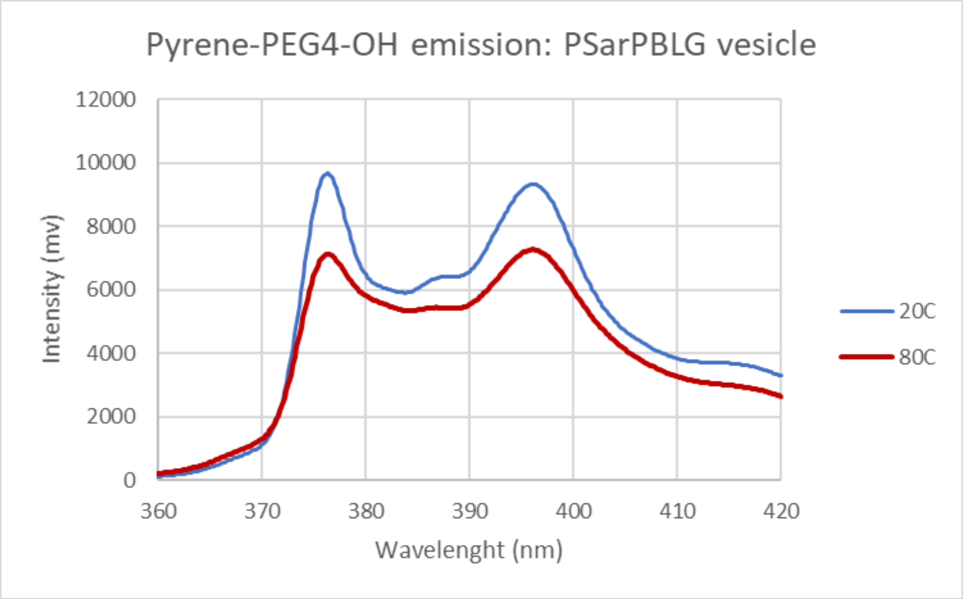

***Figure S41**: The fluorescent spectra of pyrene in the prescence of PSar-PBLG vesicles at 20℃ and 80℃ having peaks at 376.2 nm and 387.6 nm respectively.*

Critical micelle concentration

The critical micelle concentration was determined for $PBLG_{40}$-b-$PSar_{50}$ with the help of NanoSight. A dilution series was prepared from a solution of 4 mg of polymer in 1 mL of MilliQ. Starting from the lowest concentration the particle count was measured and plotted in Figure S42. The intersection of the lines was calculated to be between the concentrations 0.044 and 0.10 mg/mL.

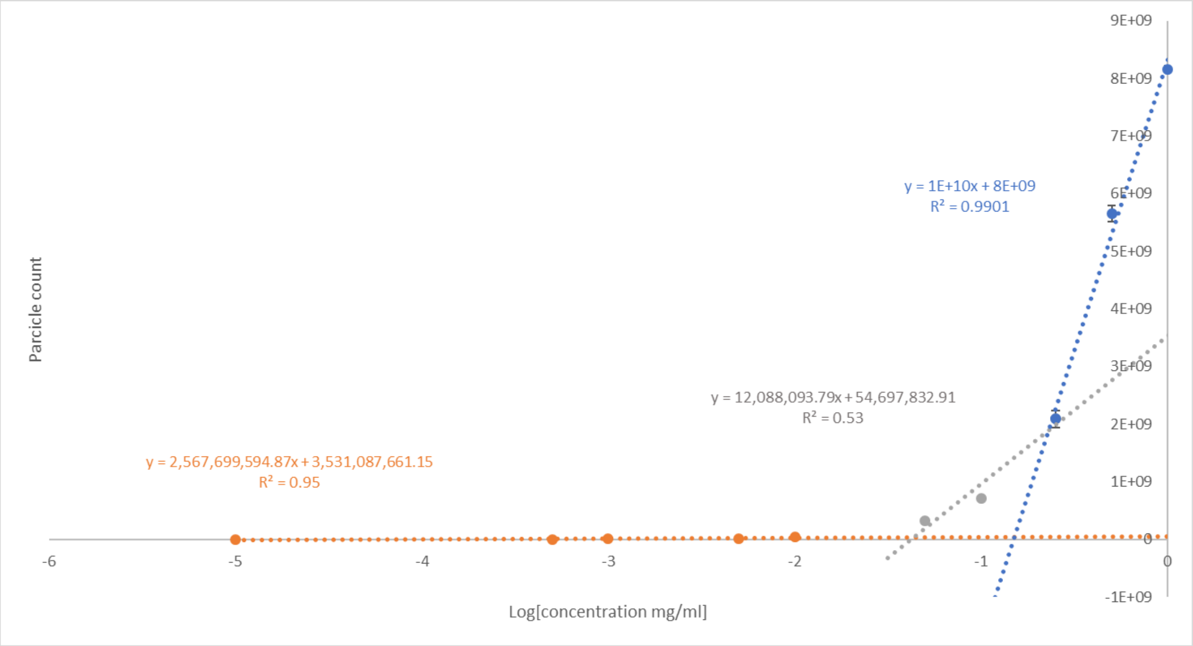

***Figure S42**: The plotted of critical micel concentration for $PBLG_{40}$-b-$PSar_{50}$, with on the y axis particle count and on the x axis the log of the polymer concentration.*

9. Stability and degradation study

To function as drug delivery vehicles, vesicles should be both biocompatible and biodegradable[4]. The speed at which the degradation occurs is of high importance for drug delivery vehicles. If the vesicles are degraded too quickly, the cargo will be released too soon and will not reach the target of interest in the body[4b]. Additionally, for biomedical applications it is desirable that vesicles have a decent shelf-life and can be stored either at room temperature (rt), in the fridge or in the freezer for future use[5]. Therefore, we set out to study the stability of $PBLG_{40}$-b-$PSar_{50}$ vesicles under different conditions.

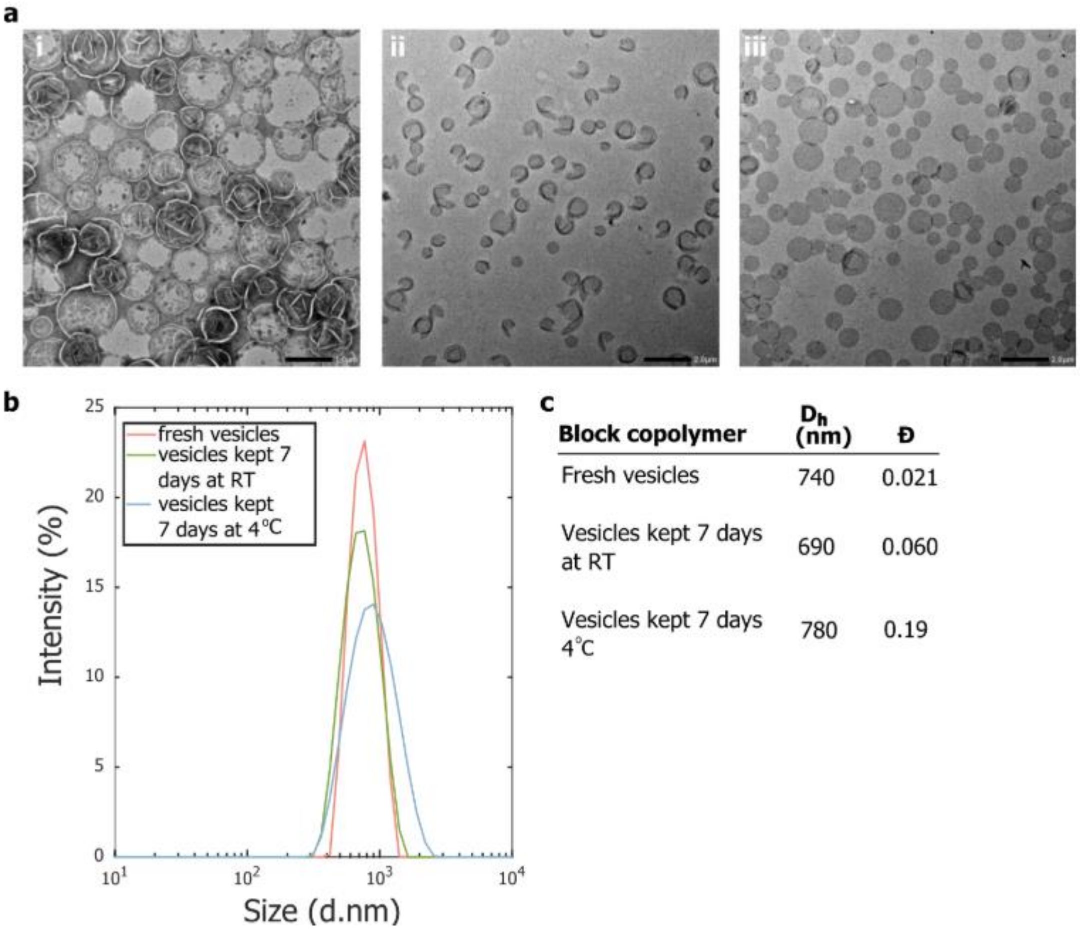

Block copolymer	D_h (nm)	Đ
Fresh vesicles	740	0.021
Vesicles kept 7 days at RT	690	0.060
Vesicles kept 7 days 4°C	780	0.19

Figure S43: Stability of PBLG40-b-PSar50 vesicles in MQ water. a) TEM images of i) freshly prepared vesicles, ii) vesicles stored at room temperature for 7 days, and iii) vesicles stored at 4 ºC for 7 days. For all TEM samples PTA was used as negative stain. b) Size distributions by DLS of vesicles kept under different conditions. c) Table of characteristics of vesicles kept at different temperatures determined by DLS.

9.1 Stability of $PBLG_{40}$-b-$PSar_{50}$ vesicles

To study the stability of $PBLG_{40}$-b-$PSar_{50}$ vesicles and examine its shelf-life, one batch of vesicles was assembled from DMF and stored at different temperatures for up to 7 days in MQ water. When kept at rt for 7 days, it was observed that the vesicles displayed a different morphology than the freshly prepared vesicles (Figure S43-i,ii). After 7 days at rt the vesicles seem more ruptured and disintegrated compared to the freshly prepared vesicles (Figure S43a-i,ii). Furthermore, it was also observed that the vesicles shrink in size compared to the freshly prepared vesicles by ≈ 50 nm (Figure S43a-ii, c). Additionally, it was determined that the dispersity of the vesicles had also increased compared to the freshly prepared vesicles (Figure S43c). Interestingly, for the vesicles that were stored at 4 °C for 7 days, a limited change in morphology was observed compared to the freshly prepared vesicles (Figure S43) However, an increase in size of ≈ 40 nm was observed, and an increase in dispersity compared to the freshly prepared vesicles (Figure S43). We believe that the difference in morphology between the freshly prepared vesicles and the vesicles stored at 4 °C on one hand, and the vesicles stored at rt on the other hand, is due to the difference in solubility of the polymer at different temperatures in water [6].

At rt, the solubility of the polymer in water is expected to be larger than at 4 °C. The increased solubility of the polymer at rt could lead to a decreased stability of the vesicle leading disintegration of the vesicle. Moreover, it was expected that the increase in size that was observed for vesicles stored at 4 °C compared to the fresh vesicles, is due to water uptake of the vesicles over time. The reason of this phenomenon is not observed for vesicles stored at rt, could be the result of the disintegration of the vesicles, which would increase the permeability, but at the same time phase out the membrane pressure. This would lead to no size increase of the vesicles.

The apparent decrease in size of the vesicles stored at rt could be due to DLS measurement errors, as DLS works suboptimal for non-spherical particles [7]. As can be seen from the cryoTEM images of the $PBLG_{40}$-b-$PSar_{50}$ vesicles, these vesicles are not perfectly spherical even when freshly prepared (Figure S19). The permeability of the vesicles at different temperatures must be quantified in order to confirm this notion. This could be achieved by incorporation of a dye (e.g. Nile red) into the vesicles and quantifying the diffusion of the dye for vesicles at different temperatures through (confocal) microscopy.

9.2. Three-week stability study

Inspired by the one-week stability study, a new batch of vesicles and also a batch of stomatocytes were prepared and stored at 4 different temperatures. These particles were monitored with DLS and TEM for 21 days. The vesicles were stored in the fridge at a temperature of 4 °C, at room temperature (≈ 20 °C), at 37 °C and lastly at 70 °C.

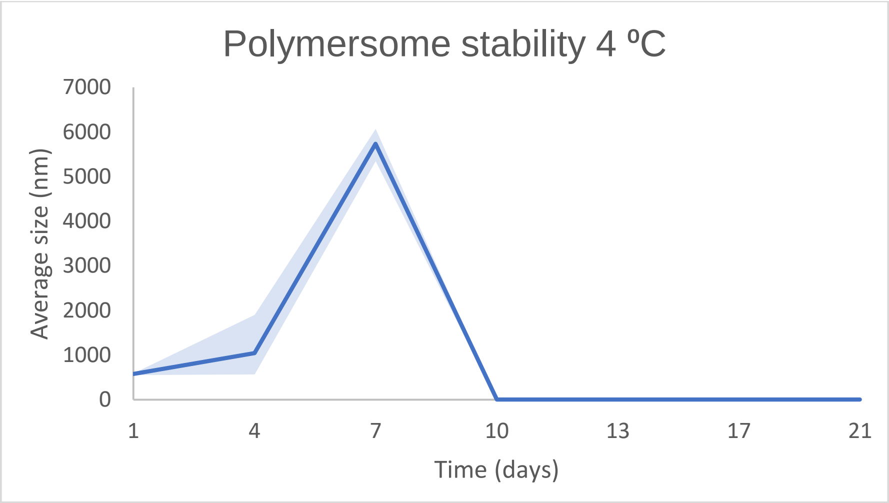

Figure S44: *DLS stability study of $PBLG_{40}$-b-$PSar_{50}$ polymersomes over 21 days at 4 ºC. After 10 days, no more polymersomes are observed.*

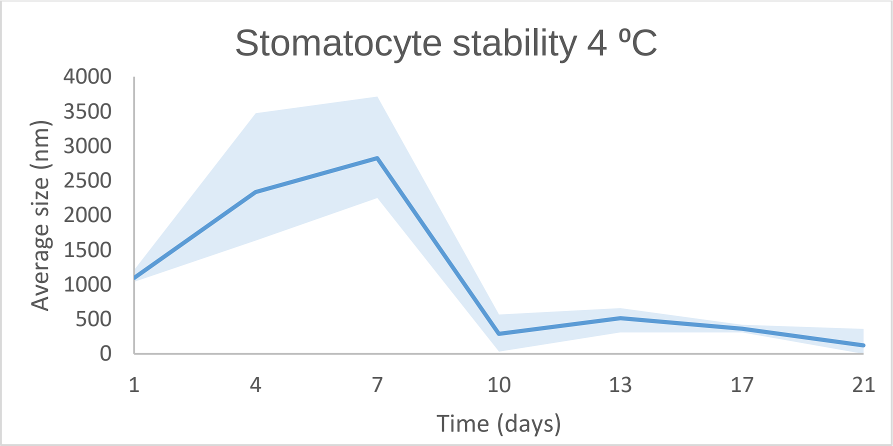

Figure S45: *DLS stability study of PBLG$_{40}$-b-PSar$_{50}$ stomatocytes over 21 days at 4 ºC. The stomatocytes show a large size distribution and the average size goes down drastically after 7 days.*

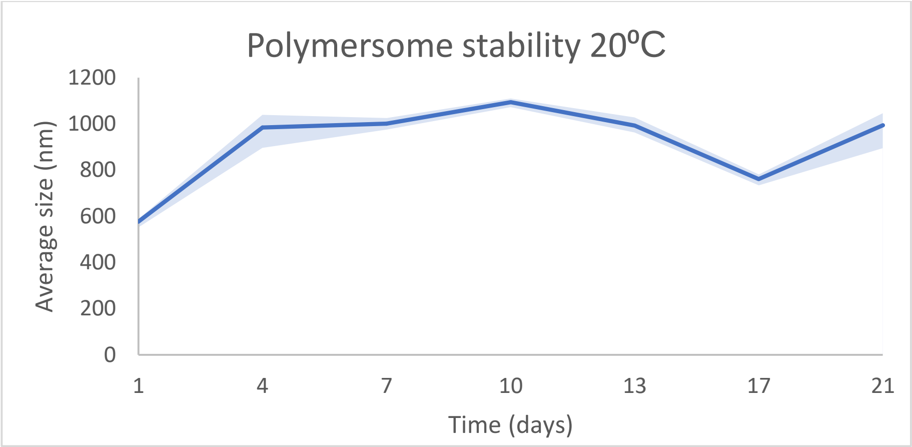

Figure S46: *DLS stability study of PBLG$_{40}$-b-PSar$_{50}$ polymersomes over 21 days at 20 ºC. The polymersomes appear to show good stability over the full duration of the experiment.*

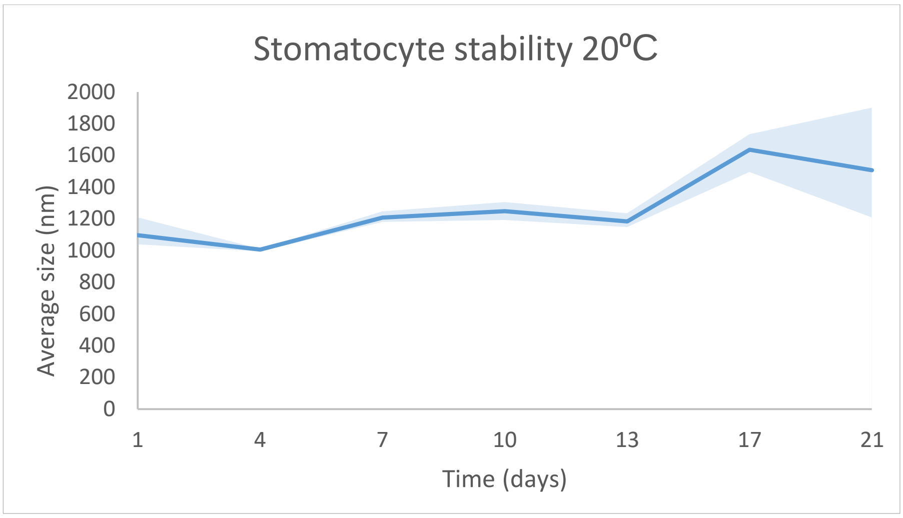

Figure S47: *DLS stability study of PBLG$_{40}$-b-PSar$_{50}$ stomatocytes over 21 days at 20 ºC. The stomatocytes appear to show good stability up to 17 days, after this there is an increase in size distribution.*

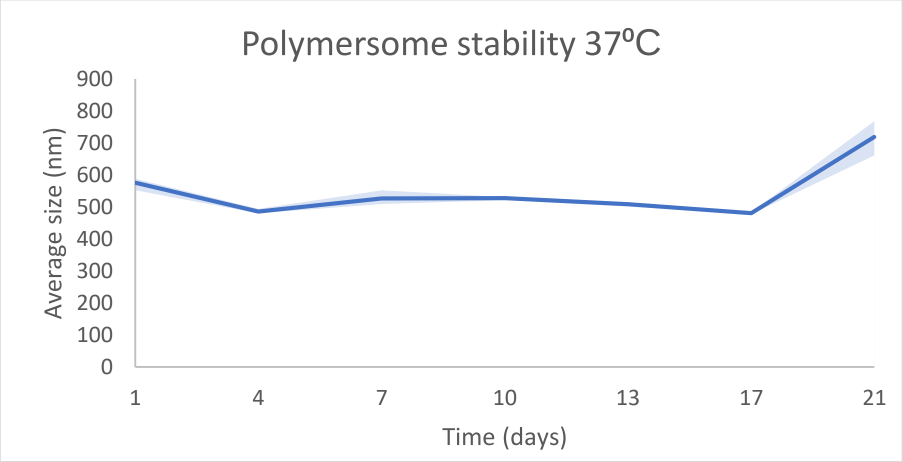

Figure S48: *DLS stability study of PBLG$_{40}$-b-PSar$_{50}$ polymersomes over 21 days at 37 ºC. The polymersomes appear to show good stability up to 17 days.*

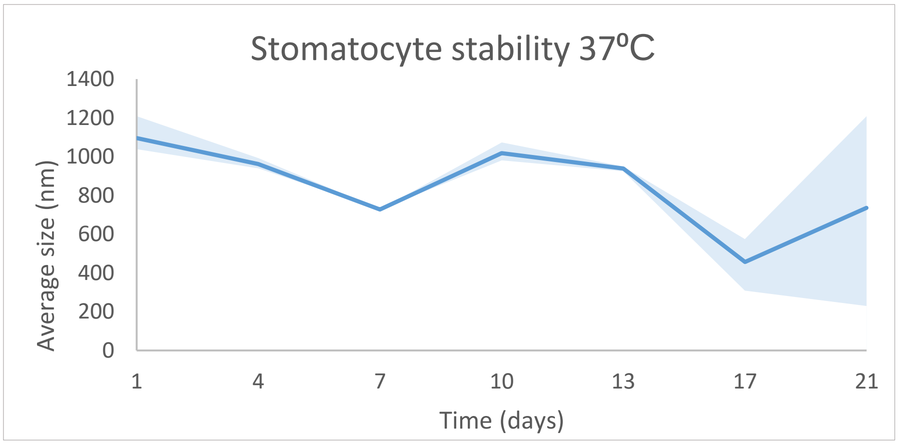

Figure S49: *DLS stability study of PBLG$_{40}$-b-PSar$_{50}$ stomatocytes over 21 days at 37 ºC. The stomatocytes appear to show good stability at the start. However, at 17 days the size distribution greatly increases.*

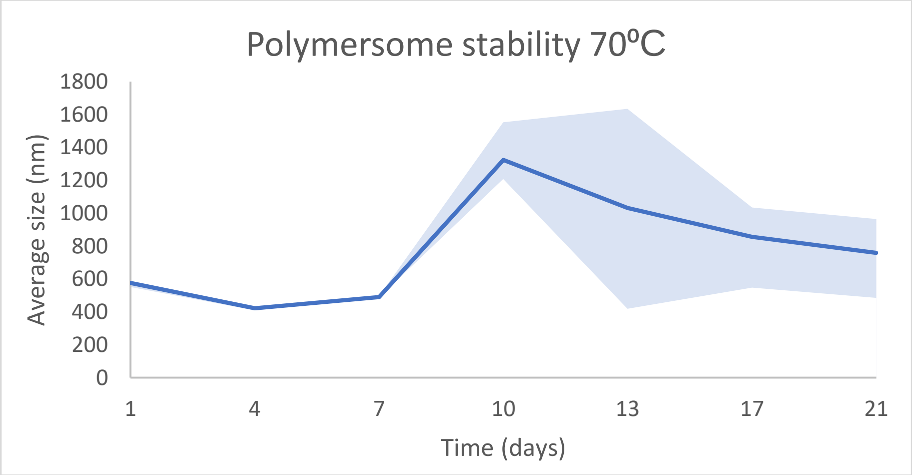

Figure S50: *DLS stability study of PBLG$_{40}$-b-PSar$_{50}$ polymersomes over 21 days at 70 ºC. A significant increase in size distribution can be observed from day 13 onwards.*

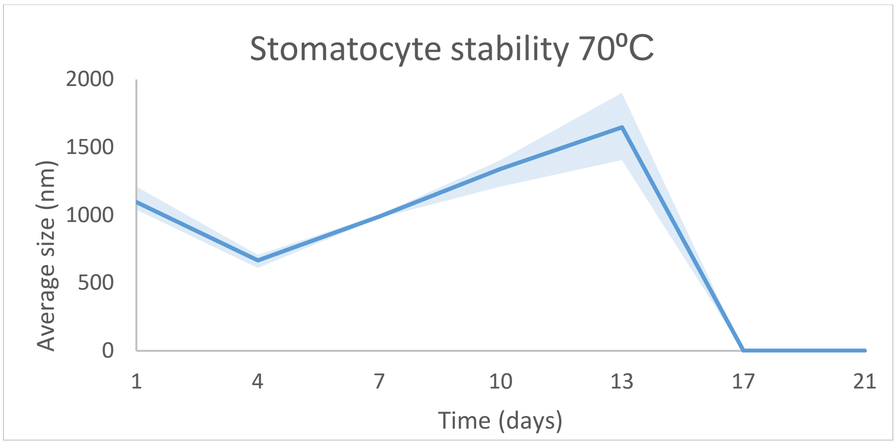

Figure S51: *DLS stability study of PBLG$_{40}$-b-PSar$_{50}$ stomatocytes over 21 days at 70 ºC. The stomatocytes appear to show good stability at the start. However, at 17 days no more stomatocytes were observed.*

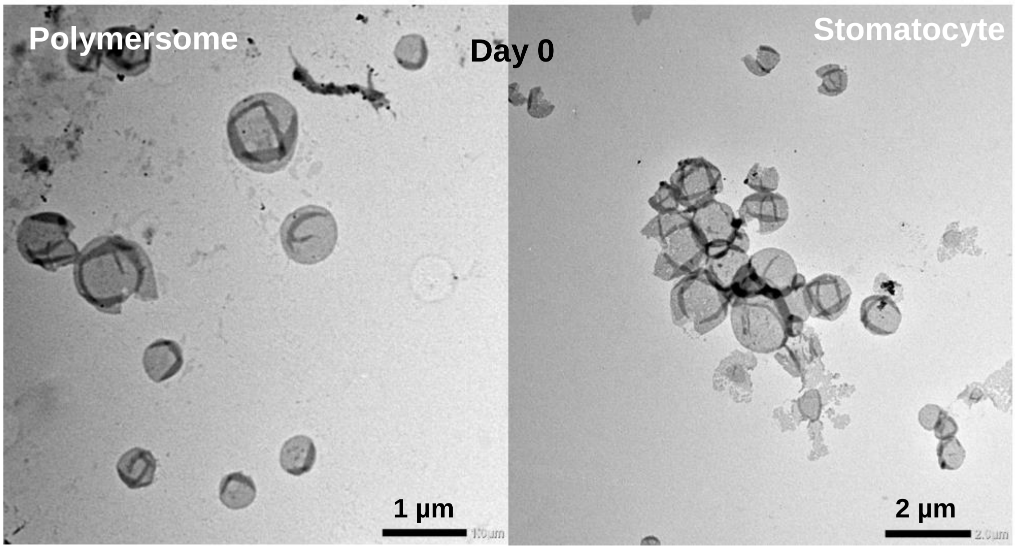

Figure S52: *TEM images of polymersomes (left) and stomatocyte (right) at the start of the stability study. PTA staining was used.*

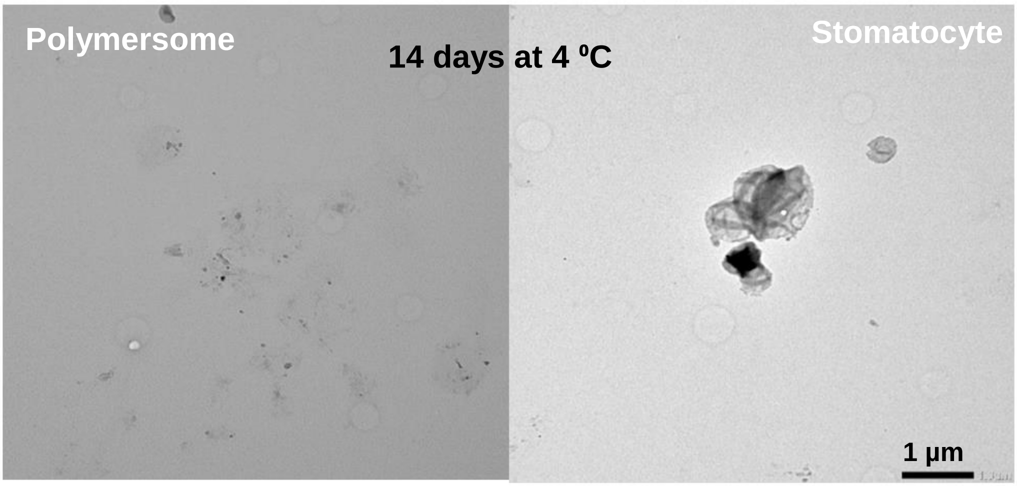

Figure S53: *TEM images of polymersomes (left) and stomatocytes (right) that were stored at 4 ºC. Images were taken after 14 days. Very few vesicles could be seen. PTA staining was used.*

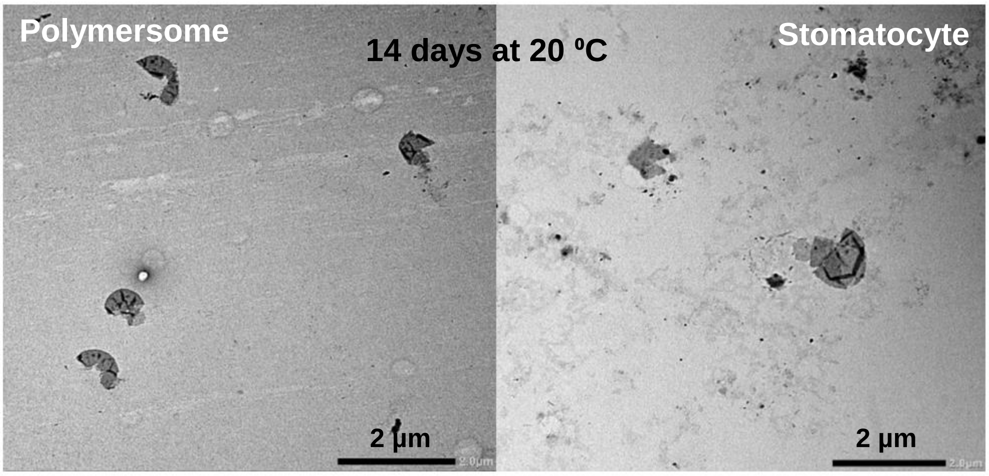

Figure S54: *TEM images of polymersomes (left) and stomatocytes (right) that were stored at 20 ºC. Images were taken after 14 days. Many vesicles were observed. PTA staining was used.*

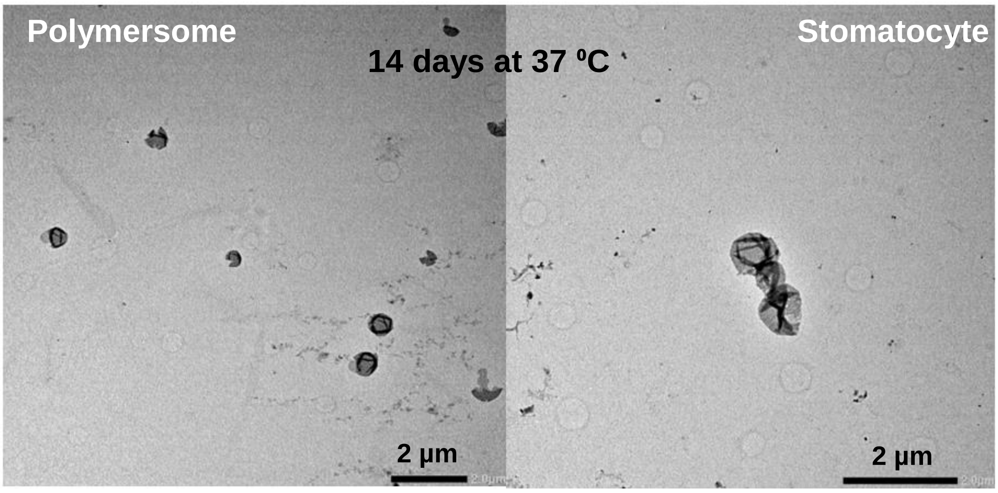

Figure S55: *TEM images of polymersomes (left) and stomatocytes (right) that were stored at 37 ºC. Images were taken after 14 days. Many vesicles were observed. PTA staining was used.*

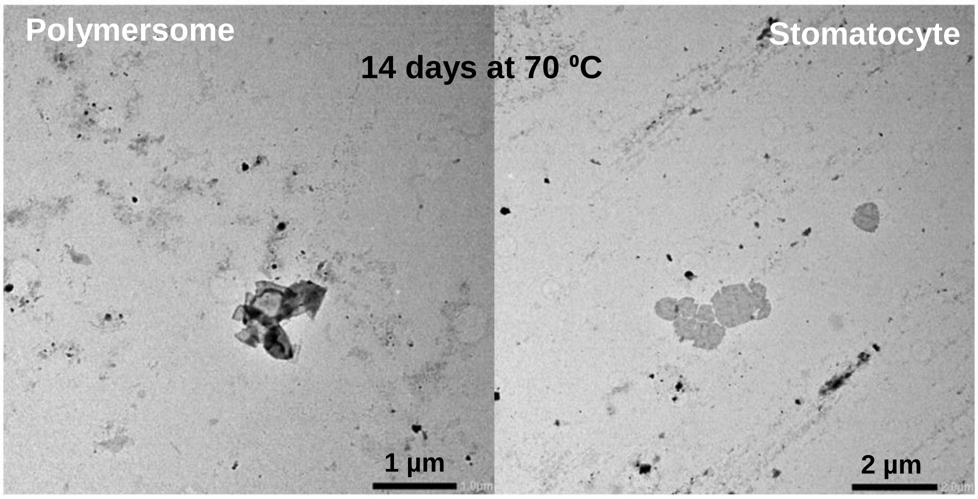

Figure S56: *TEM images of polymersomes (left) and stomatocytes (right) that were stored at 37 ºC. Images were taken after 14 days. Few vesicles were observed. PTA staining was used.*

From the DLS and TEM results it can be seen that $PBLG_{40}$-b-$PSar_{50}$ vesicles can be stored at any temperature up to 7 days, with minimal disintegration and no morphological changes. For longer storage, room temperature and 37 °C seem to give the most stability to the vesicles in terms of size and concentration of intact vesicles. It could be observed from TEM imaging that the number of vesicles that could be found during the imaging went down with longer storage.

Overall, we expect that $PBLG_{40}$-b$PSar_{50}$ vesicles provide a promising stepping stone to towards the facile fabrication of degradable vesicles for biomedical applications with a decent shelve-life.

9.3. Degradability of $PBLG_{40}$-b-$PSar_{50}$ vesicles

Over the last few years, amphiphilic polypeptides have received considerable attention for use as a new class of nanocarriers for drug delivery[8] due to their exceptional degradability [9] and biocompatibility [10].

Polypeptides have been extensively shown to have low immunogenicity, low cytotoxicity, and biodegradability leading to their attraction for biomaterials research.[11] It has been shown in literature that PBLG containing polypeptides show biodegradable properties when incubated in PBS (pH 7.4) in the presence and absence of trypsin at 37 °C [12] and in the enzymatic presence of Pseudomonas lipase. [13]

A similar class to polypeptides is polypeptoids. While polypeptides have a chiral side chain on their backbone α-carbon, polypeptoids have an achiral side chain on their amide nitrogen. This leads to a lower protease susceptibility and subsequent lower biodegradability. These properties are favourable for applications that require sustained resistance times, such as drug delivery.

Because of the achirality of poly(sarcosine), the polypeptoid is more resistant to protease, more stable and degrades slower. The substitution on the amide nitrogen also limits hydrogen bonding between the polymers, which enhances the solubility of the polymer in water and alters the chain conformation. The achirality aids in the exhibition of disordered structures, in contrast to peptidic α-helices and β-sheets.

Poly(sarcosine) is a non-ionic and highly hydrophilic poly(amino acid) or polypeptoids has shown many advantages when compared to PEG, e.g., high water solubility, easy synthesis and functionality, and neglect immunogenicity. [14] Importantly, PSar is synthesized from endogenous substances, rendering it extremely safe for human use [15]. Meanwhile, PSar is able to be degraded under acid or enzymatic environment [14a].

10. References

[1] Rasouli, M., *Clinical biochemistry* **2016**, *49*, 936-941.

[2] Lim, H. W. G.; Wortis, M., Mukhopadhyay, R., *Proc Natl Acad Sci U S A* **2002**, *99*, 16766-16769.

[3] Zhang, S.; Srivastava, A.; Li, W.; Rijpkema, S. J.; Carnevale, V.; Klein, M. L., Wilson, D. A., *J Am Chem Soc* **2023**, *145*, 10458-10462.

[4] aHerrmann, I. K.; Wood, M. J. A., Fuhrmann, G., *Nature nanotechnology* **2021**, *16*, 748-759; bWilson, D. A.; Nolte, R. J., Van Hest, J. C., *Nature chemistry* **2012**, *4*, 268-274.

[5] Peng, F.; Tu, Y., Wilson, D. A., *Chemical Society Reviews* **2017**, *46*, 5289-5310.

[6] Castro, F. L. A. d.; Campos, B. B.; Bruno, K. F., Reges, R. V., *Journal of Applied Oral Science* **2013**, *21*, 157-162.

[7] Levin, A. D.; Shmytkova, E. A., Khlebtsov, B. N., *The Journal of Physical Chemistry C* **2017**, *121*, 3070-3077.

[8] Stevens, C. A.; Kaur, K., Klok, H.-A., *Advanced Drug Delivery Reviews* **2021**, *174*, 447-460.

[9] Liu, Z.; Dong, C.; Wang, X.; Wang, H.; Li, W.; Tan, J., Chang, J., *ACS applied materials & interfaces* **2014**, *6*, 2393-2400.

[10] Vandermeulen, G. W., Klok, H. A., *Macromolecular Bioscience* **2004**, *4*, 383-398.

[11] Hutchinson, F., Furr, B., *Journal of Controlled Release* **1990**, *13*, 279-294.

[12] aTian, H.; Deng, C.; Lin, H.; Sun, J.; Deng, M.; Chen, X., Jing, X., *Biomaterials* **2005**, *26*, 4209-4217; bLi, L.; Shi, X.; Wang, Z.; Wang, Y.; Jiao, Z., Zhang, P., *Journal of Materials Chemistry B* **2018**, *6*, 3315-3330.

[13] Le Hellaye, M.; Fortin, N.; Guilloteau, J.; Soum, A.; Lecommandoux, S., Guillaume, S. M., *Biomacromolecules* **2008**, *9*, 1924-1933.

[14] aBirke, A.; Ling, J., Barz, M., *Progress in Polymer Science* **2018**, *81*, 163-208; bAlberg, I.; Kramer, S.; Schinnerer, M.; Hu, Q.; Seidl, C.; Leps, C.; Drude, N.; Möckel, D.; Rijcken, C., Lammers, T., *Small* **2020**, *16*, 1907574; cMakino, A.; Hara, E.; Hara, I.; Yamahara, R.; Kurihara, K.; Ozeki, E.; Yamamoto, F., Kimura, S., *Journal of controlled release* **2012**, *161*, 821-825; dWeber, B.; Kappel, C.; Scherer, M.; Helm, M.; Bros, M.; Grabbe, S., Barz, M., *Macromolecular Bioscience* **2017**, *17*, 1700061.

[15] Yao, X.; Qi, C.; Sun, C.; Huo, F., Jiang, X., *Nano Today* **2023**, *48*, 101738.
